# Author Correction: A single residue in the yellow fever virus envelope protein modulates virion architecture and antigenicity

**DOI:** 10.1038/s41467-025-65890-x

**Published:** 2025-11-05

**Authors:** Summa Bibby, James Jung, Yu Shang Low, Alberto A. Amarilla, Natalee D. Newton, Connor A. P. Scott, Jessica Balk, Yi Tian Ting, Morgan E. Freney, Benjamin Liang, Timothy Grant, Fasséli Coulibaly, Paul Young, Roy A. Hall, Jody Hobson-Peters, Naphak Modhiran, Daniel Watterson

**Affiliations:** 1 School of Chemistry & Molecular Biosciences, The University of Queensland, St Lucia, QLD, Australia; 2 Australian Infectious Disease Research Centre, The University of Queensland, St Lucia, QLD Australia; 3 Department of Biochemistry and Molecular Biology and Biomedicine Discovery Institute, Monash University, Clayton, VIC, Australia; 4 John and Jeanne Rowe Center for Research in Virology, Morgridge Institute for Research, Madison, WI USA; 5 Department of Biochemistry, University of Wisconsin-Madison, Madison, WI USA; 6 Present Address: Janelia Research Campus, Howard Hughes Medical Institute, Ashburn, VA USA; 7 Present Address: Centre for Inflammatory Diseases, Department of Medicine, School of Clinical Sciences, Monash University, Clayton, VIC Australia

**Keywords:** Cryoelectron microscopy, Virus structures

Correction to: *Nature Communications* 10.1038/s41467-025-63038-5, published online 26 September 2025

The original version of this article contained an error in the Data Availability Statement. The accession number for bYFV-Asibi particle is corrected from EMD-44904 to EMD-49804.

This has now been amended in the HTML and PDF versions of the article.

