# Supplementary material for: A single residue in the yellow fever virus envelope protein modulates virion architecture and antigenicity
Source: Nat Commun. 2025 Sep 26;16:8449. doi: 10.1038/s41467-025-63038-5 (PMC12475062; doi:10.1038/s41467-025-63038-5)
Supplement: Supplementary file 1 — Supplementary Information [file 41467_2025_63038_MOESM1_ESM.pdf]

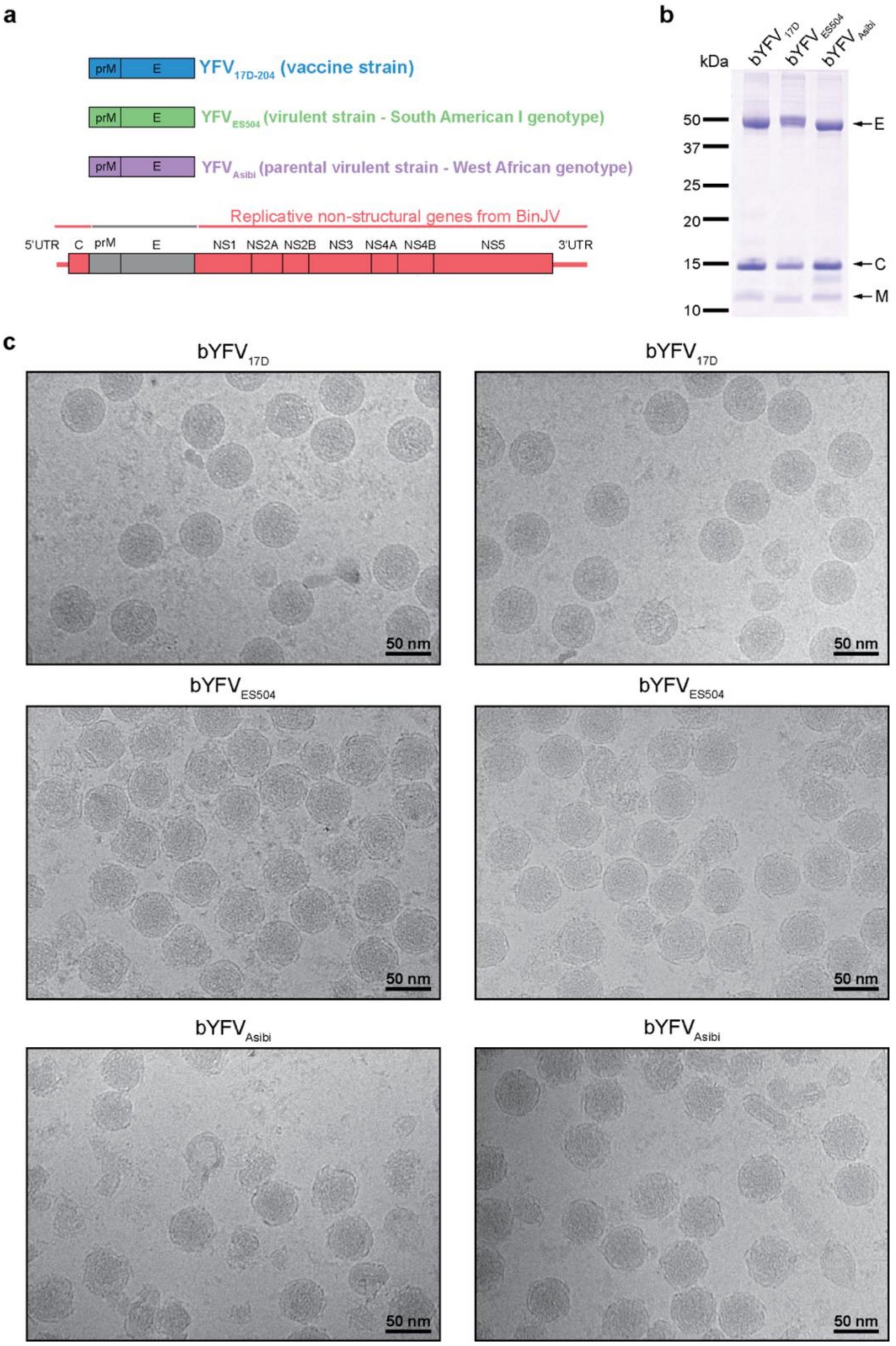

Supplementary Fig. 1: SDS-PAGE and representative micrographs of bYFVs. a Schematic of the bYFV genomes. **b** Purified viruses (10 μg) were separated by SDS-PAGE under reducing conditions and stained with Coomassie Blue. Viral protein identity indicated by arrows. **c** Representative cryo-EM micrographs of bYFV$_{17D}$ and bYFV$_{ES504}$ incubated at 4°C prior to freezing. The representative micrographs are indicative of two independent biological replicates. Source data has been provided as a Source Data file.

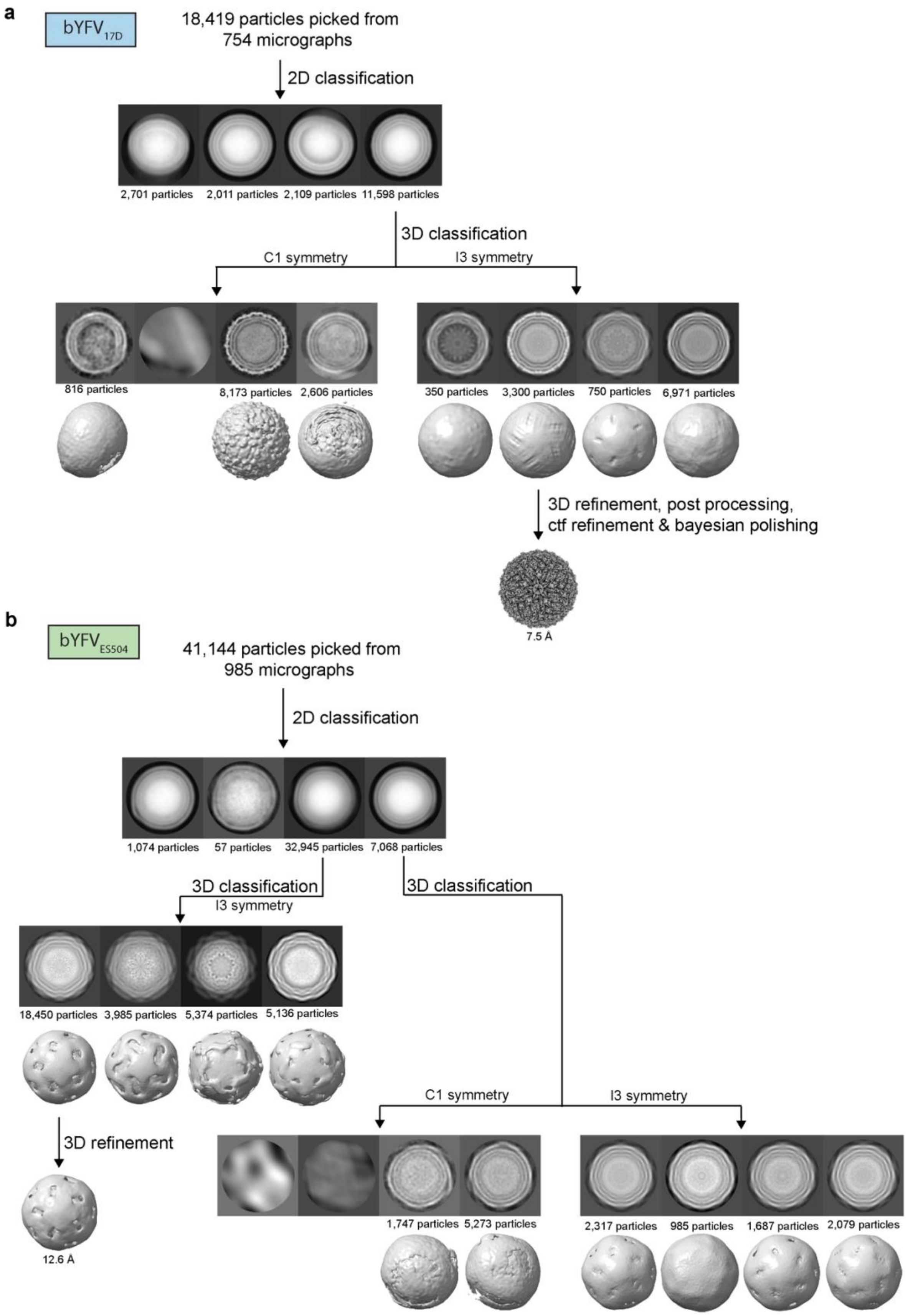

Supplementary Fig. 2: Cryo-EM processing flowchart of bYFV$_{17D}$ and bYFV$_{ES504}$. Flowchart of single particle analysis steps performed on RELION 3.1.3 to reach the final 3D reconstructions of bYFV$_{17D}$ (**a**) and bYFV$_{ES504}$ (**b**). Displayed resolution values were calculated with half-map FSC$_{0.143}$ criterion.

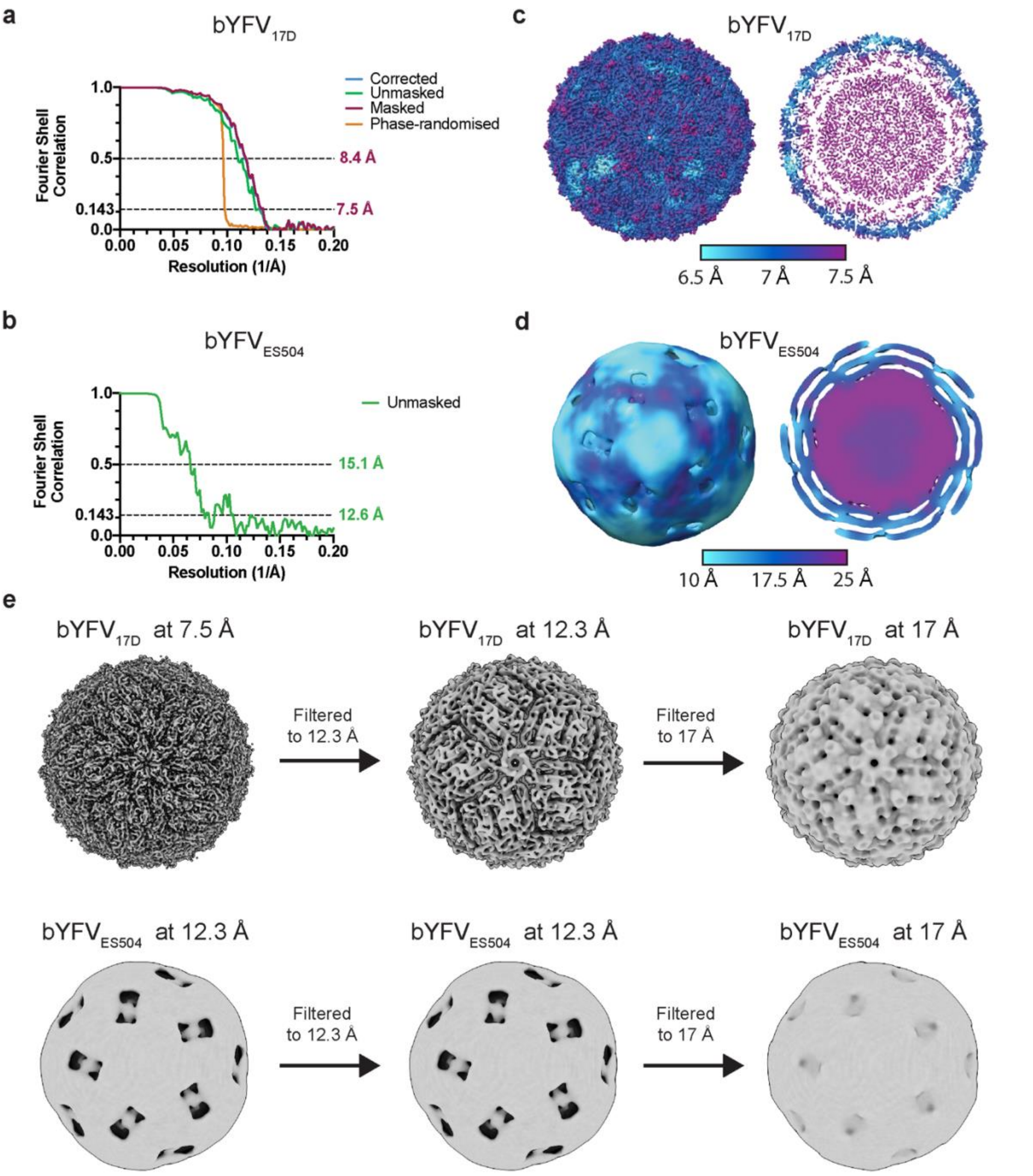

Supplementary Fig. 3: FSC curves and local resolution maps of bYFV$_{17D}$ and bYFV$_{ES504}$. FSC plots for the bYFV$_{17D}$ (**a**) and bYFV$_{ES504}$ (**b**) reconstructions. Density map of bYFV$_{17D}$ (**c**) and bYFV$_{ES504}$ (**d**) coloured according to their local resolutions. **(e)** Cryo-EM reconstructions of bYFV$_{17D}$ and bYFV$_{ES504}$ filtered at 12.3 Å and 17 Å. Source data has been provided as a Source Data file.

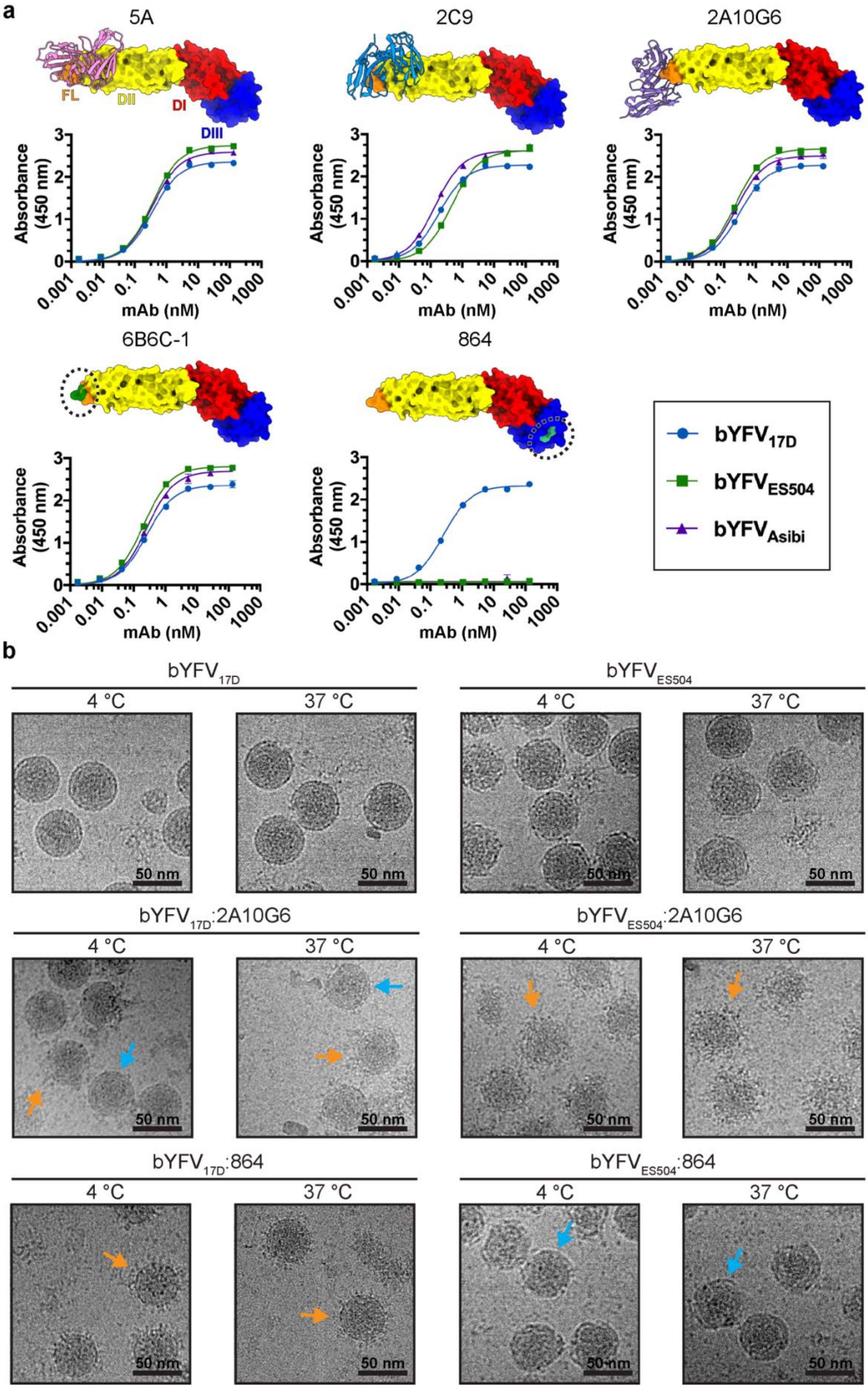

a
5A
2C9
2A10G6
FL
DII
DI
DIII
Absorbance (450 nm)
mAb (nM)
6B6C-1
864
bYFV17D
bYFVES504
bYFVAsibi
b
bYFV17D
bYFVES504
4 °C
37 °C
50 nm
bYFV17D:2A10G6
bYFVES504:2A10G6
bYFV17D:864
bYFVES504:864

Supplementary Fig. 4: Epitopes of anti-YFV and anti-flavivirus mAbs and their reactivity against bYFV$_{17D}$, bYFV$_{ES504}$ and bYFV$_{Asibi}$. a Top, the binding epitopes of 5A (PDB: 6IW2), 2C9, 2A10G6 (PDB: 5JHL), 6B6C-1 and 864 mapped onto the YFV E protein. DI, DII, DIII and the FL are coloured red, yellow, blue and orange respectively. The known residues involved with the binding epitope of 6B6C-1 and 864 have been highlighted on the E protein in dark green and light green, respectively. Below, ELISA curves of the indicated recombinant mAbs against bYFV$_{17D}$, bYFV$_{ES504}$, bYFV$_{Asibi}$. **b** Representative micrographs of bYFV$_{17D}$ and bYFV$_{ES504}$ with and without complexing with 2A10G6 or 864 Fab. Viruses and Fabs were combined in a 2:1 ratio and incubated for 1 hour at either 4°C or 37°C prior to vitrification. The arrows highlight virion surface with Fab bound (orange) or with no Fab bound (blue). The representative micrographs are indicative of two independent biological replicates. Source data has been provided as a Source Data file.

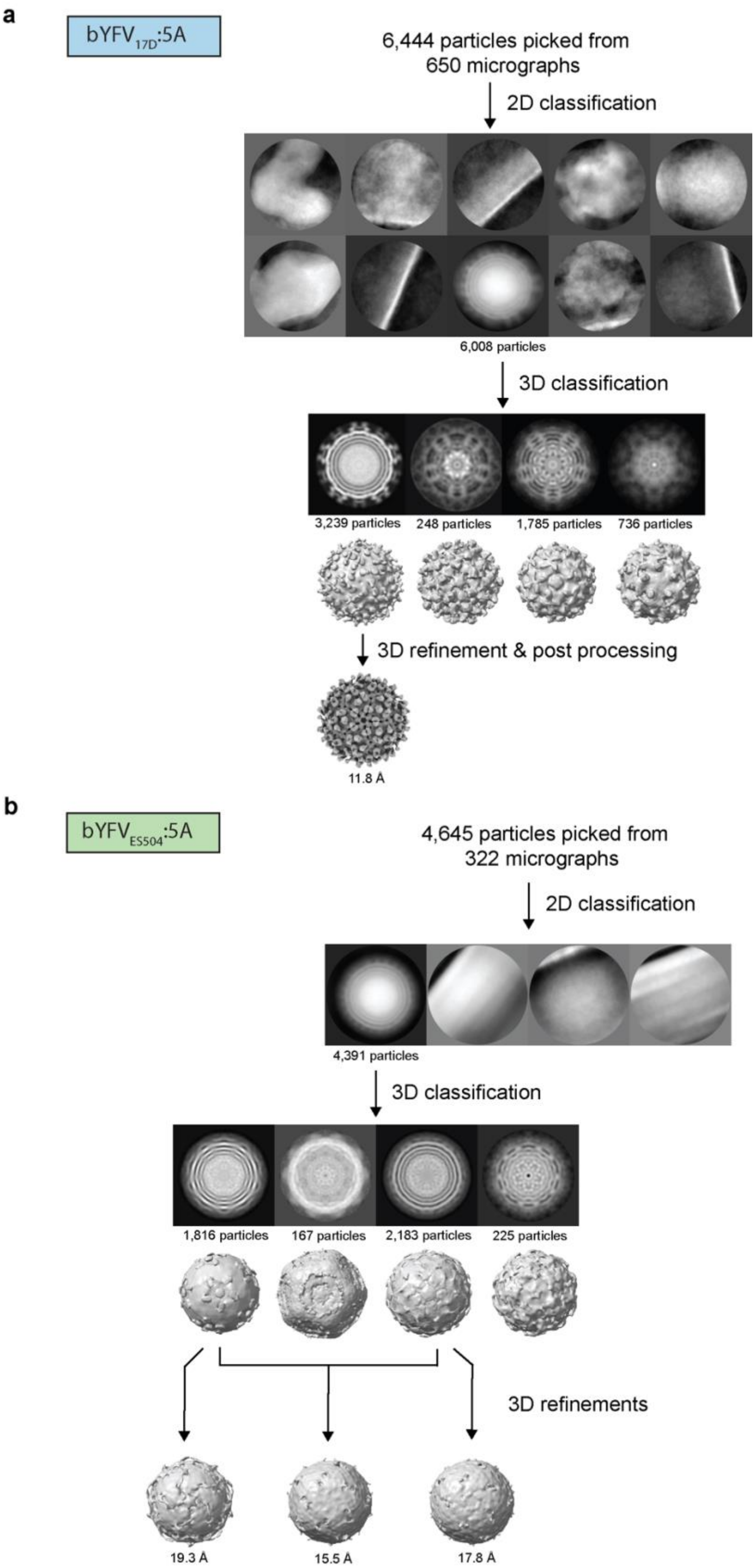

Supplementary Fig. 5: Cryo-EM processing flowcharts of bYFV$_{17D}$:5A and bYFV$_{ES504}$:5A. Flowchart of single particle analysis steps performed on RELION 3.1.3 to reach the final 3D reconstructions of bYFV$_{17D}$:5A (**a**) and bYFV$_{ES504}$:5A (**b**). Displayed resolution values were calculated with half-map FSC$_{0.143}$ criterion.

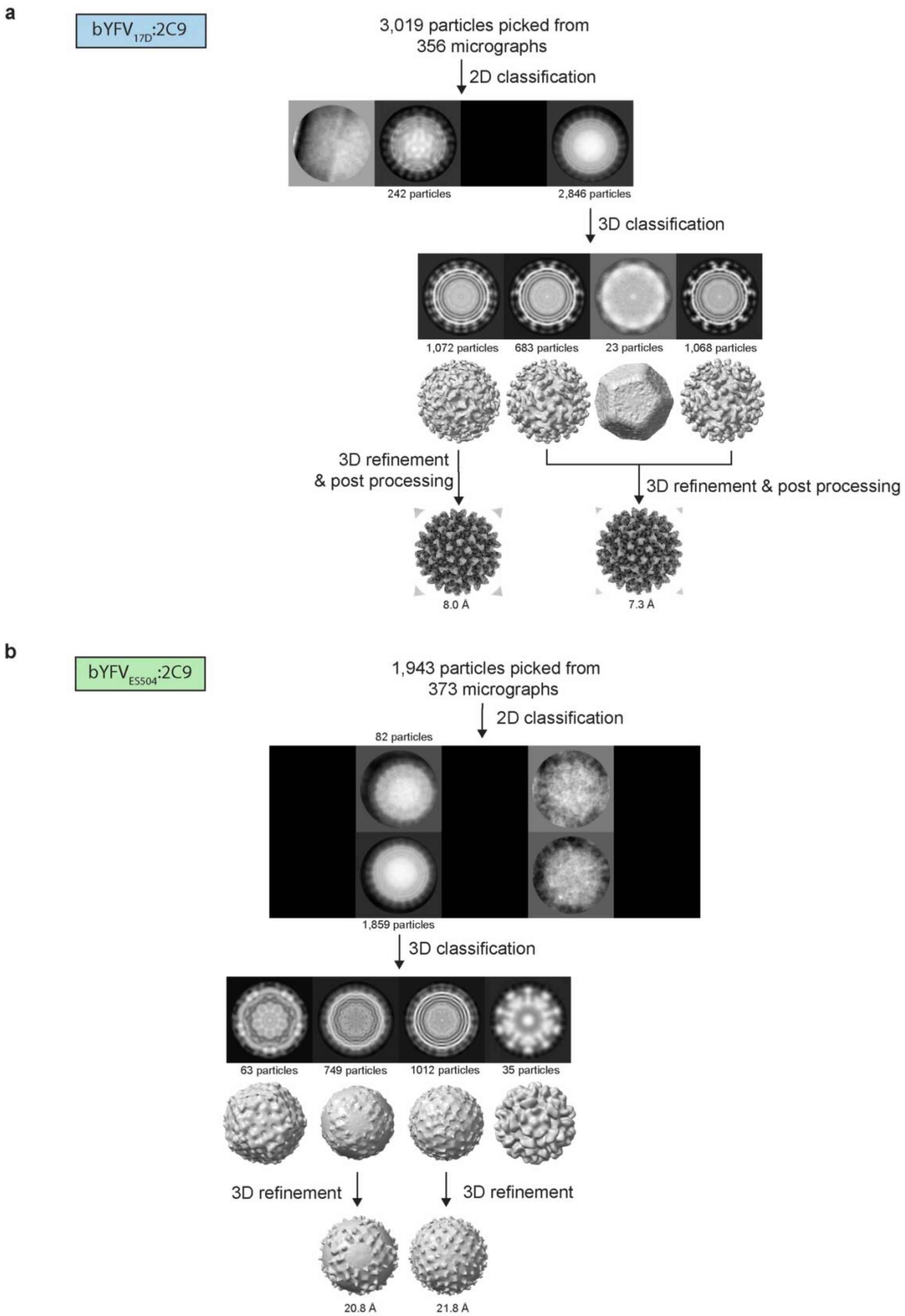

Supplementary Fig. 6: Cryo-EM processing flowcharts of bYFV$_{17D}$:2C9 and bYFV$_{ES504}$:2C9. Flowchart of single particle analysis processing steps performed on RELION 3.1.3 to reach the final 3D cryo-EM reconstructions of bYFV$_{17D}$:2C9 (**a**) and bYFV$_{ES504}$:2C9 (**b**). Displayed resolution values were calculated with half-map FSC$_{0.143}$ criterion.

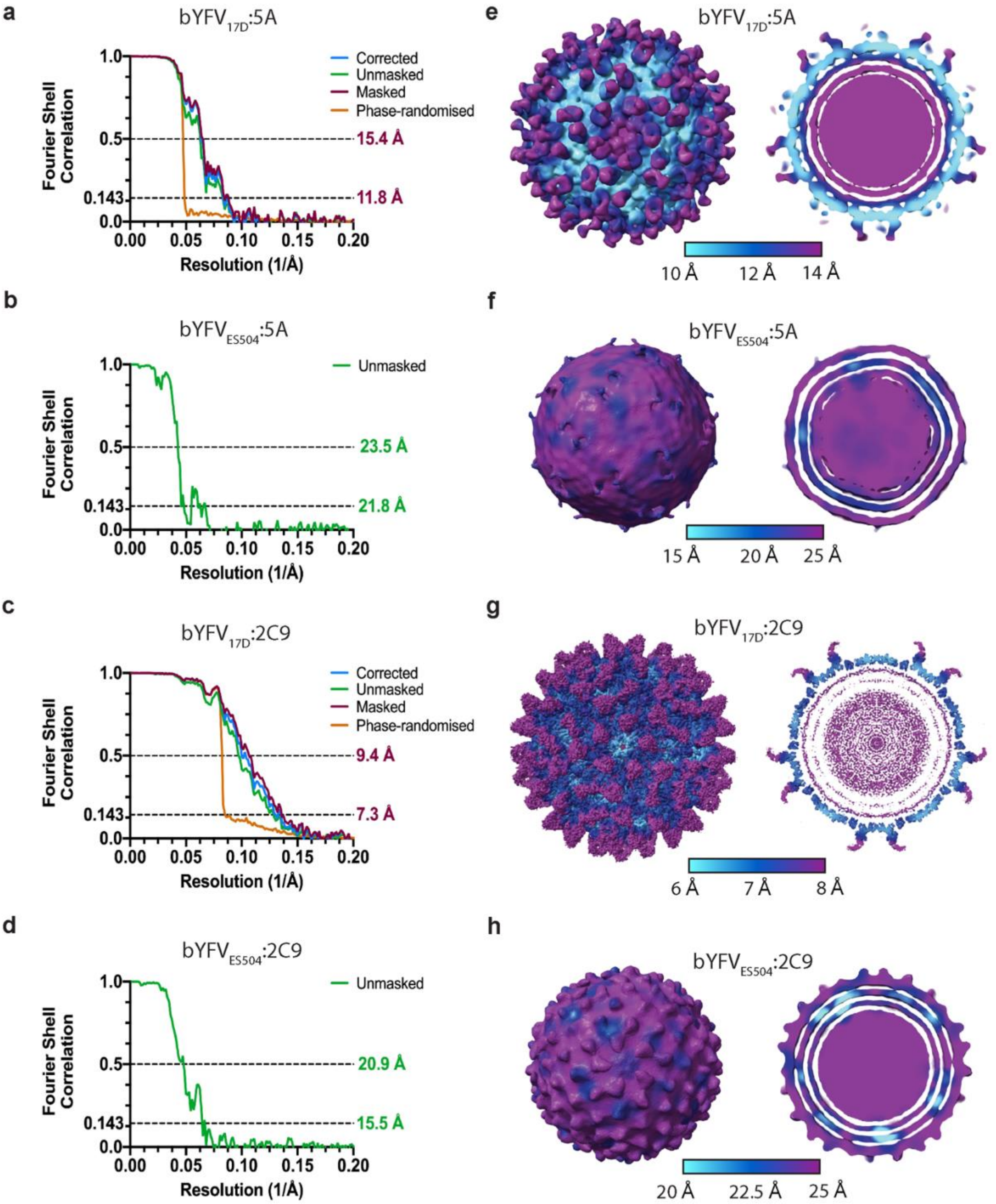

Supplementary Fig. 7: FSC curves and local resolution maps of 5A and 2C9 fab complexes with bYFV$_{17D}$ and bYFV$_{ES504}$. FSC curves for the bYFV$_{17D}$:5A (**a**), bYFV$_{ES504}$:5A (**b**), bYFV$_{17D}$:2C9 (**c**) and bYFV$_{ES504}$:2C9 (**d**) reconstructions. Density maps of bYFV$_{17D}$:5A (**e**), bYFV$_{ES504}$:5A (**f**), bYFV$_{17D}$:2C9 (**g**) and bYFV$_{ES504}$:2C9 (**h**) coloured according to their local resolution. Local resolutions were calculated on RELION 3.1.3. Source data has been provided as a Source Data file.

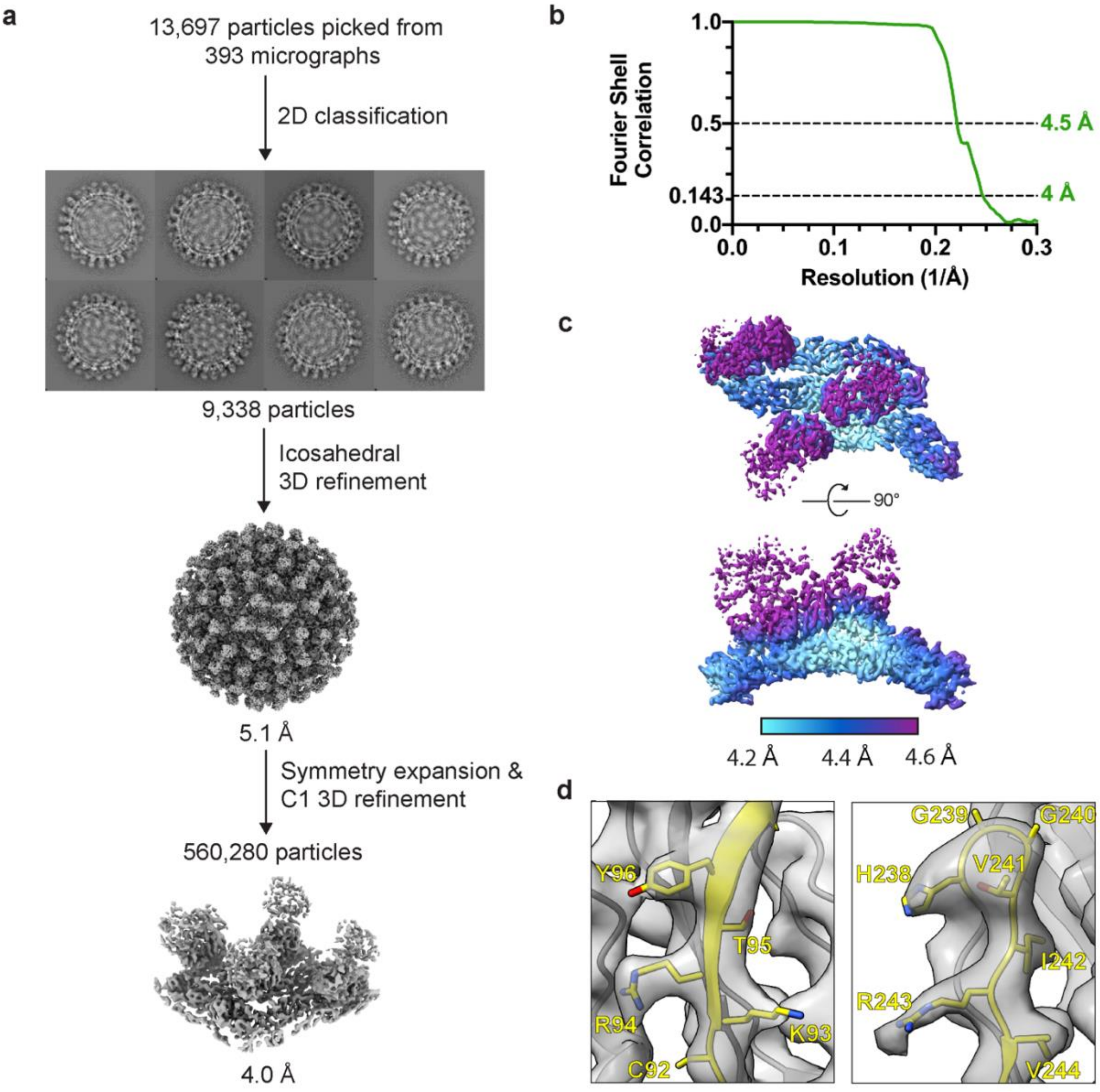

Supplementary Fig. 8: Cryo-EM processing flowchart, FSC plot and local resolution map of bYFV$_{17D}$:2C9 ASU. a Flowchart of single particle analysis performed on *cis*TEM2 to reach the final 3D cryo-EM reconstruction of bYFV$_{17D}$:2C9 ASU. Displayed resolution values were calculated with half-map FSC$_{0.143}$ criterion. **b** Part. FSC plot of bYFV$_{17D}$:2C9 ASU, calculated using a loose spherical mask on *cis*TEM2. **c** Density map of bYFV$_{17D}$:2C9 ASU coloured according to its local resolution. Local resolution values were calculated using RELION 3.1.3 with *cis*TEM2 generated half maps. **d** High resolution detail of the bYFV$_{17D}$:2C9 ASU atomic model fitted into the cryo-EM density map. Source data has been provided as a Source Data file.

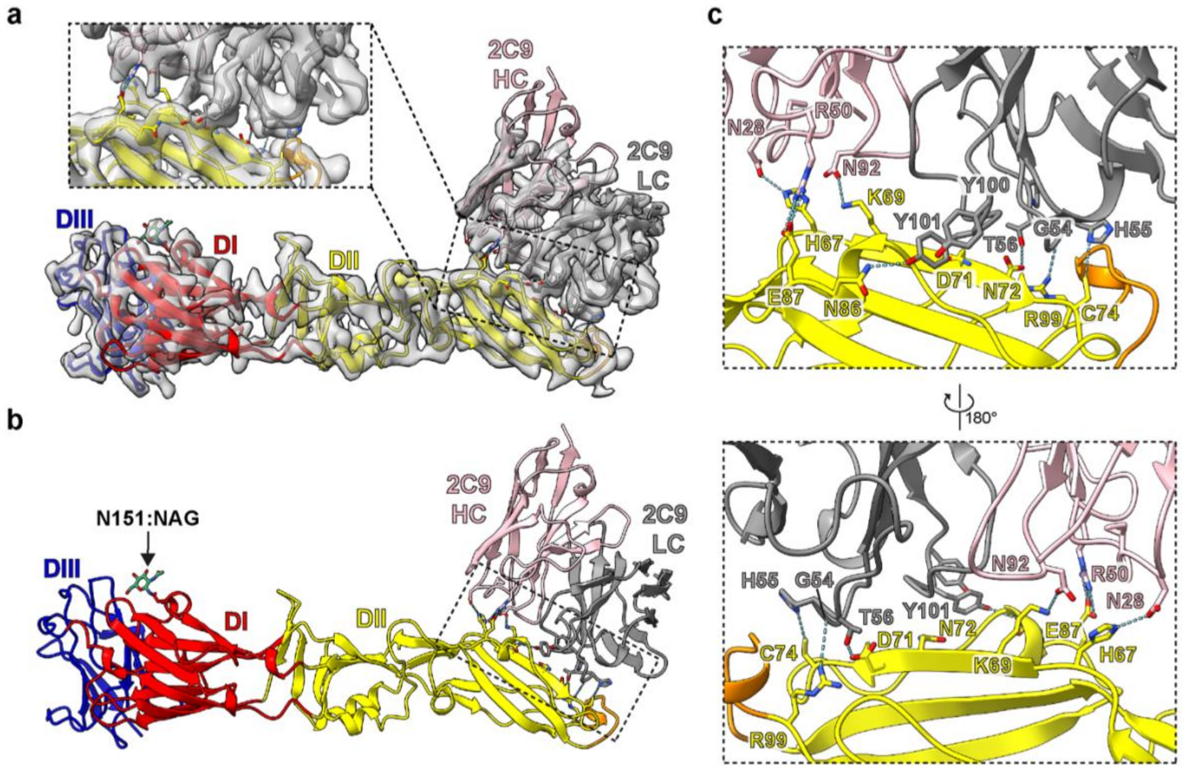

d

Donor	Acceptor	Distance
E – H67	2C9 LC – N28	3 Å
E – K69	2C9 LC – N92	3.3 Å
E – C74	2C9 HC – H55	3.6 Å
E – N86	2C9 HC – Y101	3.2 Å
E – R99	2C9 HC – G54	3.3 Å
2C9 HC – T56	E – D72	2.8 Å
2C9 LC – R50	E – E87	2.8 Å
2C9 LC – R50	E – E87	2.9 Å
2C9 LC – R50	E – E87	3.3 Å

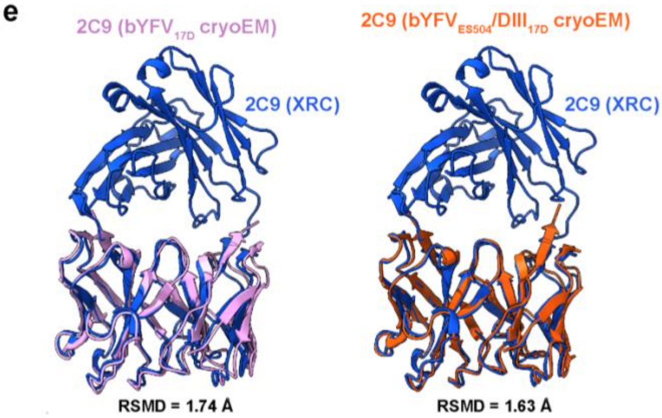

Supplementary Fig. 9: Binding epitope of mAb 2C9. a Cryo-EM density map and near-atomic model of bYFV$_{17D}$ complexed with 2C9 fab; E-DI in red, E-DII in yellow, E-DIII in blue, fusion loop in orange, 2C9 HC in grey and 2C9 LC in light pink. **b** Near-atomic model of bYFV$_{17D}$:2C9. **c** Close-up view of the bYFV$_{17D}$:2C9 model highlighting 2C9's epitope. Intermolecular bonds are shown in turquoise. **d** Summary of the intermolecular bonds present between bYFV$_{17D}$ and 2C9 fab. Intermolecular bonds were analysed using Chimera X. **e** A 2.66 Å structure of the free 2C9 Fab was determined by X-ray crystallography (XRC, blue) and shows high similarity with the 2C9 variable domain regions built from cryo-EM data of the Fab in complex with bYFV$_{17D}$ (pink, left panel) and bYFV$_{ES504}$/DIII$_{17D}$ (orange, right panel), rigid body all atom RMSD indicated. Related to Figure 2.

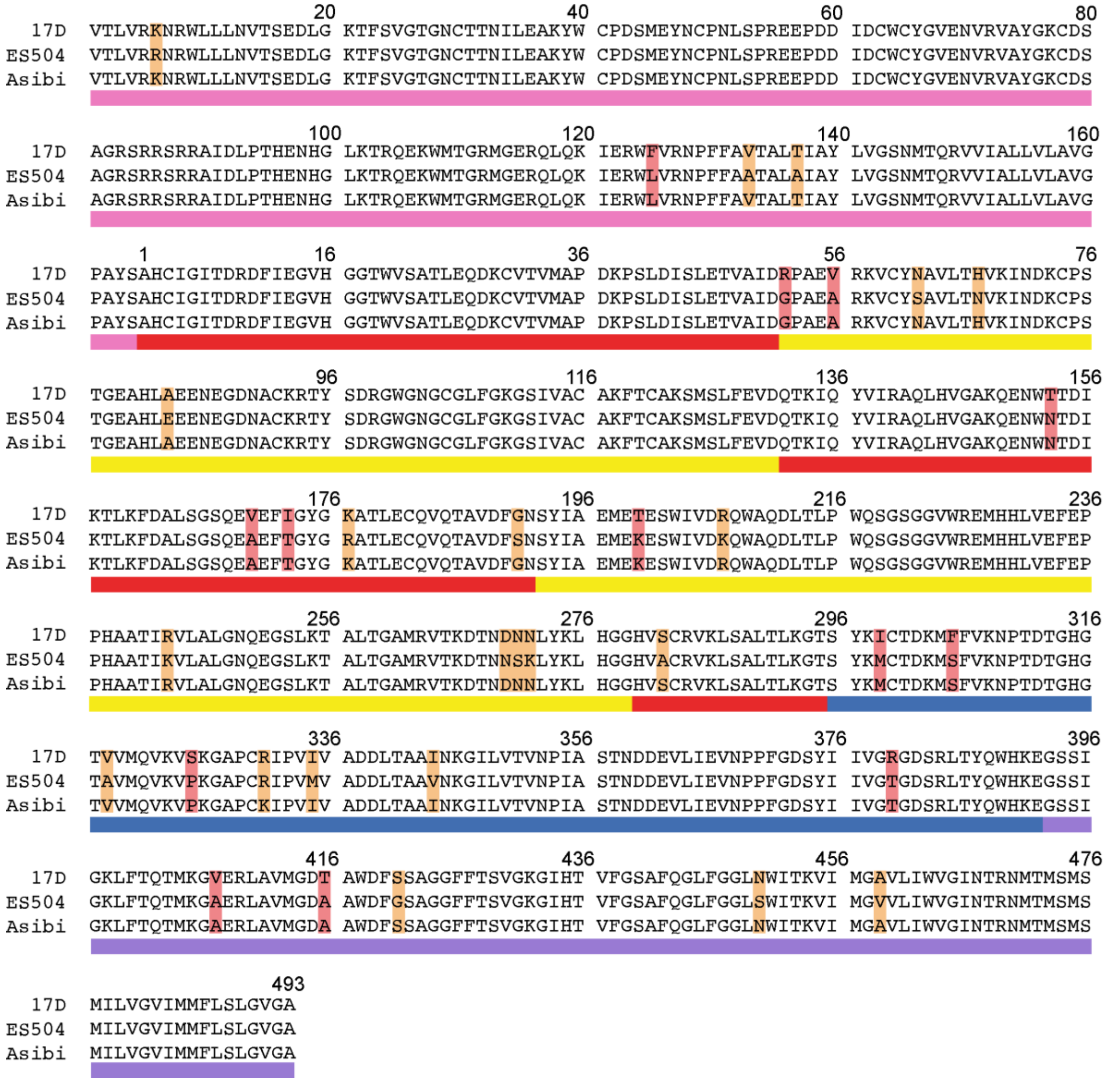

Supplementary Fig. 10: Comparison of YFV strains. Amino acid alignment of the prM and E proteins from YFV_{17D} (KF769015.1), YFV_{ES504} (KY885000.2) and YFV_{Asibi} (AY640589.1). Non-conserved amino acids are highlighted in red and orange. prM, E-DI, E-DII, E-DIII and E-TM are labelled in pink, red, yellow, blue and purple respectively.

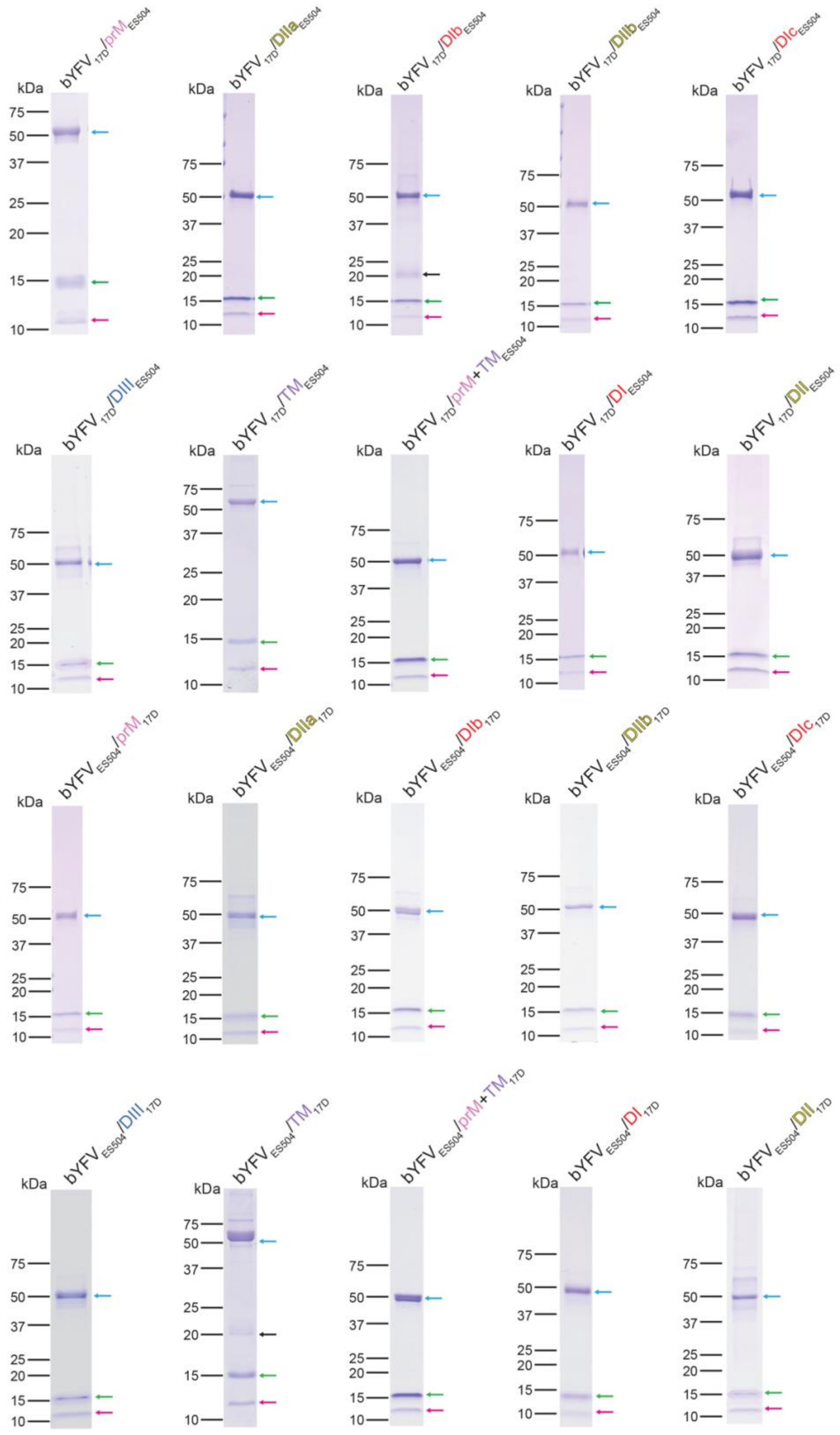

Supplementary Fig. 11: SDS-PAGE of bYFV prME chimeras. Purified viruses (10 μg) were separated by SDS-PAGE under reducing conditions and stained with Coomassie Blue. Envelope, pre-membrane, capsid and membrane proteins are indicated by blue, black, green, and pink arrows, respectively. Source data has been provided as a Source Data file.

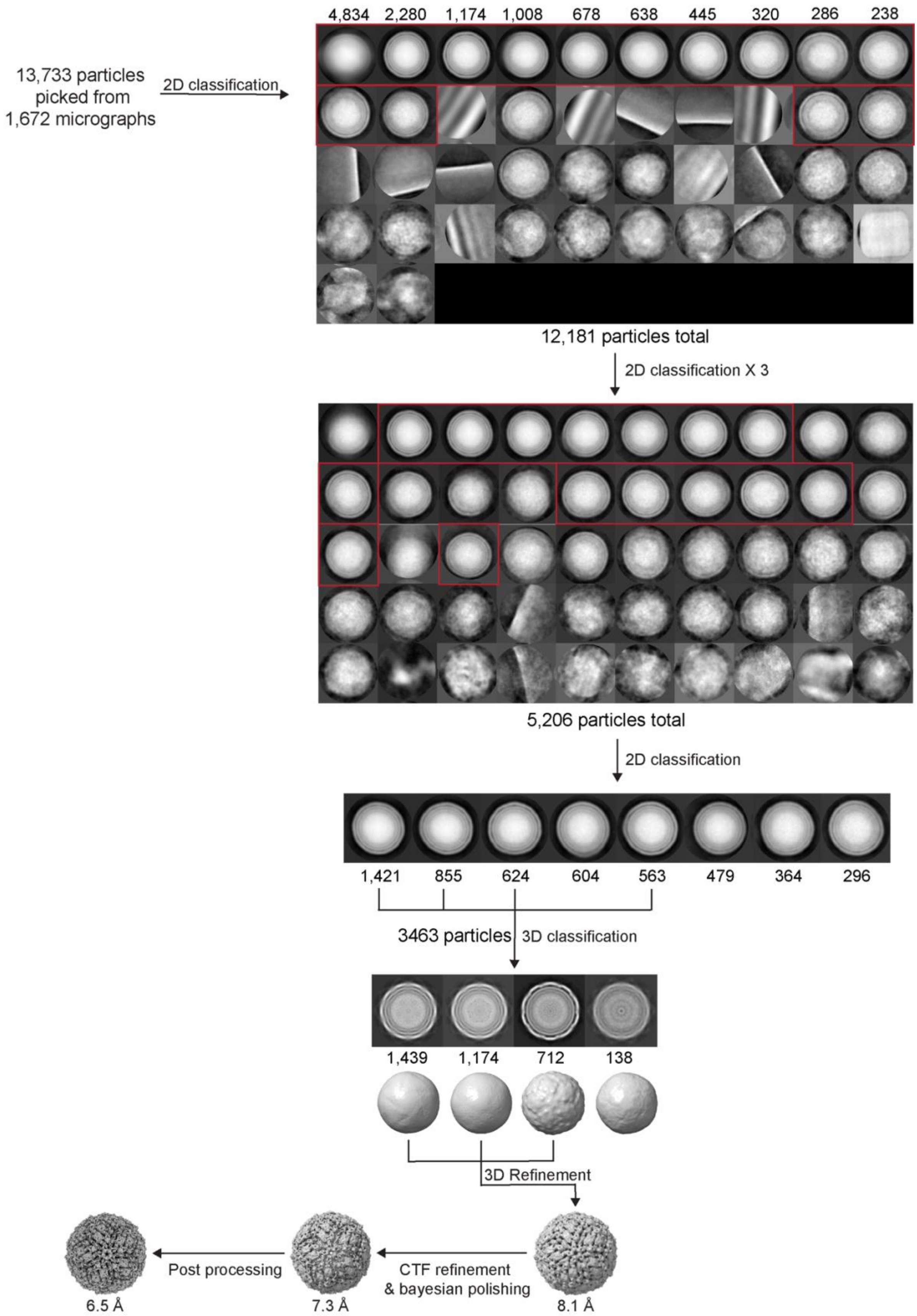

Supplementary Fig. 12: Cryo-EM processing flowchart of bYFV$_{ES504}$/DIII$_{17D}$. Flowchart of single particle analysis steps performed on RELION 3.1.3 to reach the final 3D reconstruction of bYFV$_{ES504}$/DIII$_{17D}$. Displayed resolution values were calculated with half-map FSC$_{0.143}$ criterion.

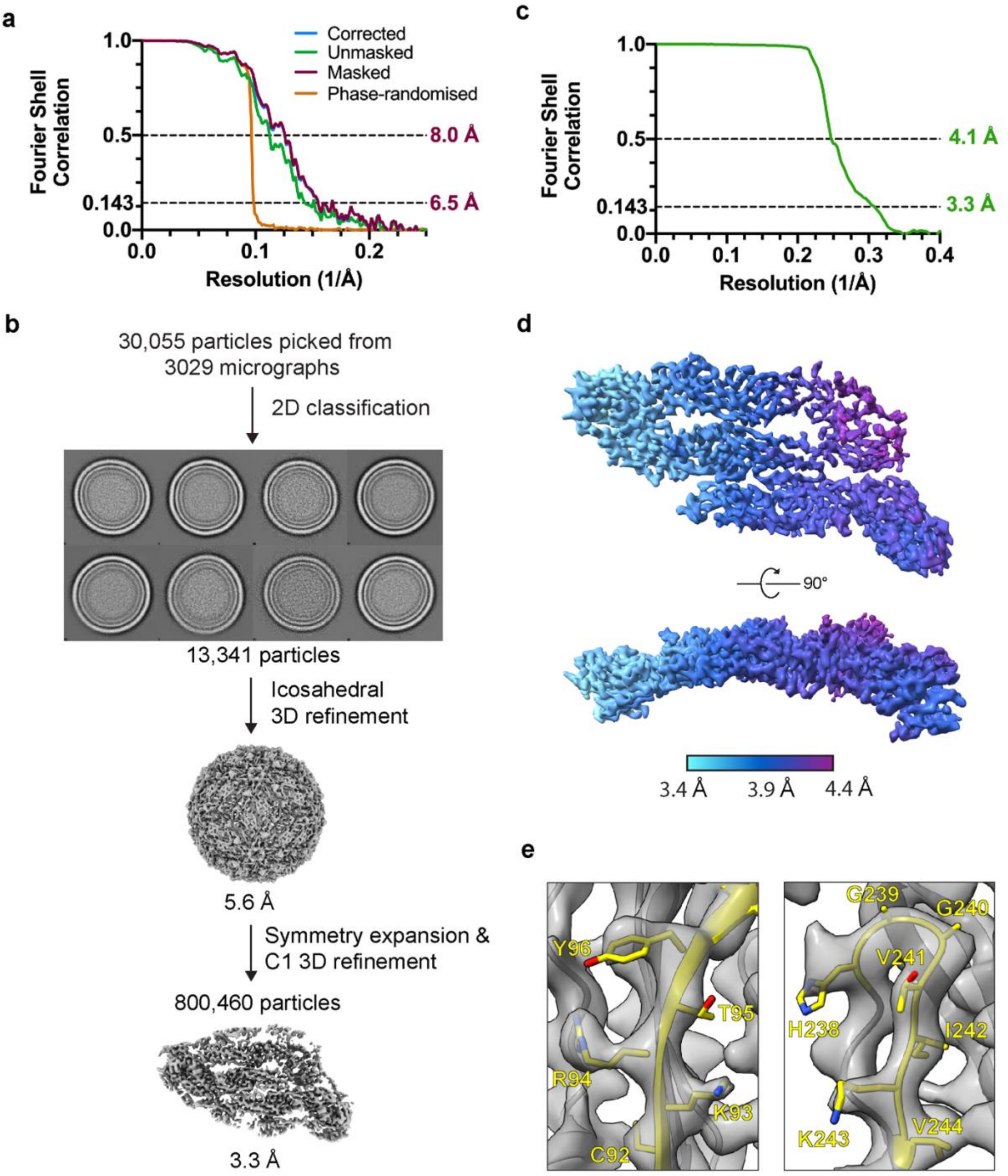

Supplementary Fig. 13: Cryo-EM processing details of the bYFV$_{ES504}$/DIII$_{17D}$ reconstructions. **a** FSC plot of the bYFV$_{ES504}$/DIII$_{17D}$ reconstruction. **b** Flowchart of single particle analysis performed on *cis*TEM2 to reach the final cryo-EM reconstruction of bYFV$_{ES504}$/DIII$_{17D}$ ASU. Displayed resolution values were calculated with half-map FSC$_{0.143}$ criterion. **c** Part. FSC plot of bYFV$_{ES504}$/DIII$_{17D}$ ASU, calculated using a loose spherical mask on *cis*TEM2. **d** Density map of bYFV$_{ES504}$/DIII$_{17D}$ ASU coloured according to its local resolution. Local resolution values were calculated using RELION 3.1.3 with *cis*TEM2 generated half maps. **e** High resolution detail of the bYFV$_{ES504}$/DIII$_{17D}$ ASU atomic model fitted in the cryo-EM density map. Source data has been provided as a Source Data file.

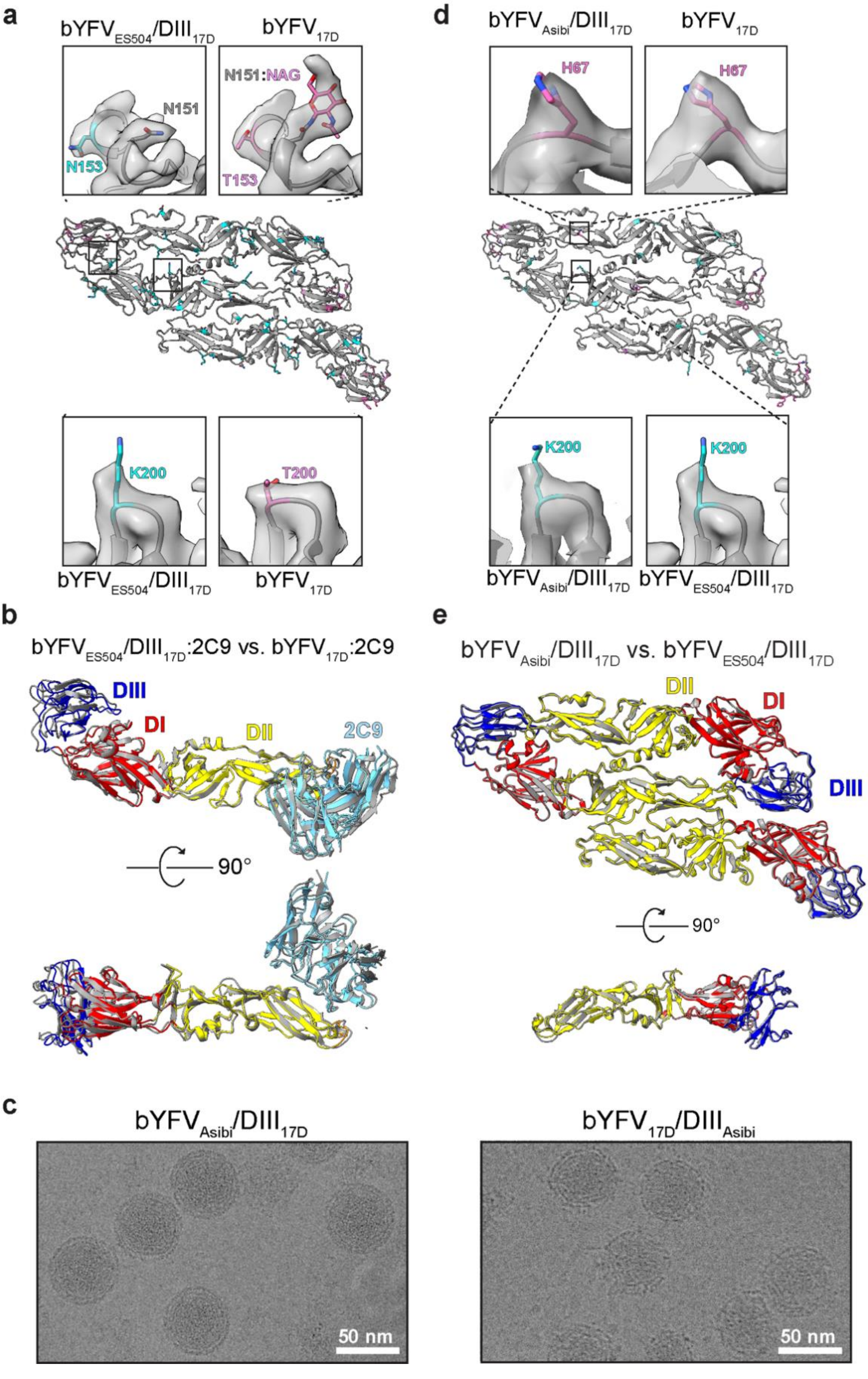

a
bYFV$_{ES504}$/DIII$_{17D}$
bYFV$_{17D}$
N151
N153
N151:NAG
T153
K200
T200
bYFV$_{ES504}$/DIII$_{17D}$
bYFV$_{17D}$
d
bYFV$_{Asibi}$/DIII$_{17D}$
bYFV$_{17D}$
H67
H67
K200
K200
bYFV$_{Asibi}$/DIII$_{17D}$
bYFV$_{ES504}$/DIII$_{17D}$
b
bYFV$_{ES504}$/DIII$_{17D}$:2C9 vs. bYFV$_{17D}$:2C9
DIII
DI
DII
2C9
90°
e
bYFV$_{Asibi}$/DIII$_{17D}$ vs. bYFV$_{ES504}$/DIII$_{17D}$
DII
DI
DIII
90°
c
bYFV$_{Asibi}$/DIII$_{17D}$
bYFV$_{17D}$/DIII$_{Asibi}$
50 nm
50 nm

Supplementary Fig. 14: Atomic models of bYFV$_{ES504}$/DIII$_{17D}$, bYFV$_{ES504}$/DIII$_{17D}$:2C9 and bYFV$_{Asibi}$/DIII$_{17D}$. **a** Atomic model of the bYFV$_{ES504}$/DIII$_{17D}$ ASU with the non-YFV$_{17D}$ amino acids highlighted in turquoise and pink. Boxes illustrate key differences between the bYFV$_{ES504}$/DIII$_{17D}$ and bYFV$_{17D}$:2C9 cryo-EM density maps. **b** Comparison of the bYFV$_{ES504}$/DIII$_{17D}$ and bYFV$_{17D}$ 5-fold E protein atomic models (Cα RMSD: 1.25). The pentagons, triangles, and ovals represent the 5-, 3- and 2-fold axes. **c** Representative micrographs of purified bYFV$_{Asibi}$/DIII$_{17D}$ and bYFV$_{17D-204}$/DIII$_{Asibi}$ virions at 4°C. **d** Atomic model of the bYFV$_{Asibi}$/DIII$_{17D}$ ASU, with the non-YFV$_{17D}$ amino acids coloured in turquoise and pink. Boxes illustrate key differences between the bYFV$_{Asibi}$/DIII$_{17D}$, bYFV$_{ES504}$/DIII$_{17D}$ and bYFV$_{17D}$:2C9 cryo-EM density maps. **e** Comparison of the bYFV$_{Asibi}$/DIII$_{17D}$ and the bYFV$_{ES504}$/DIII$_{17D}$ (in grey) atomic models, aligned at the 5-fold E protein (Cα RMSD: 0.85).

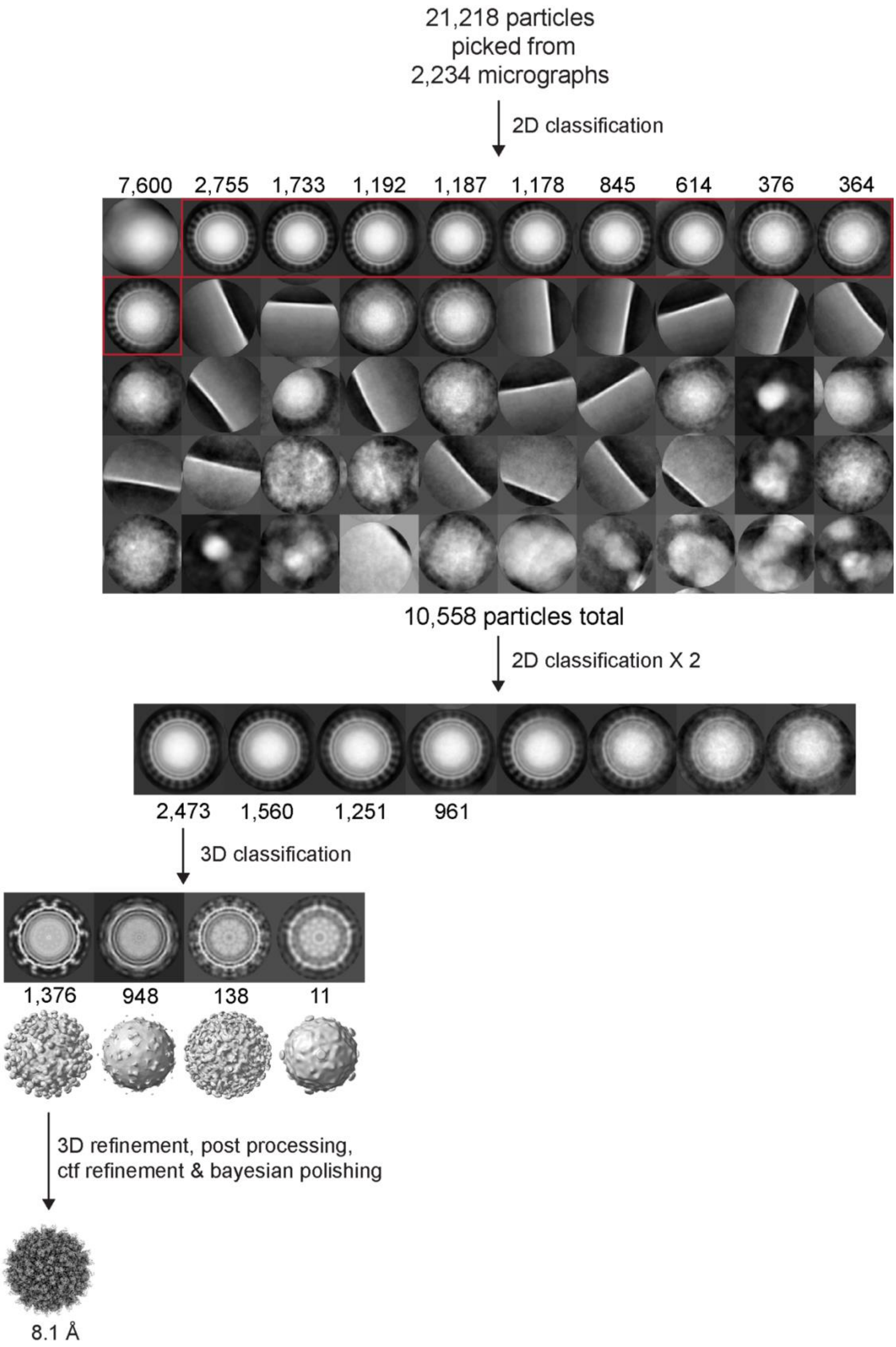

Supplementary Fig. 15: Cryo-EM processing flowchart of bYFV$_{ES504}$/DIII$_{17D}$:2C9. Flowchart of single particle analysis steps performed on RELION 3.1.3 to reach the final 3D reconstruction of bYFV$_{ES504}$/DIII$_{17D}$:2C9. Displayed resolution values were calculated with half-map FSC$_{0.143}$ criterion.

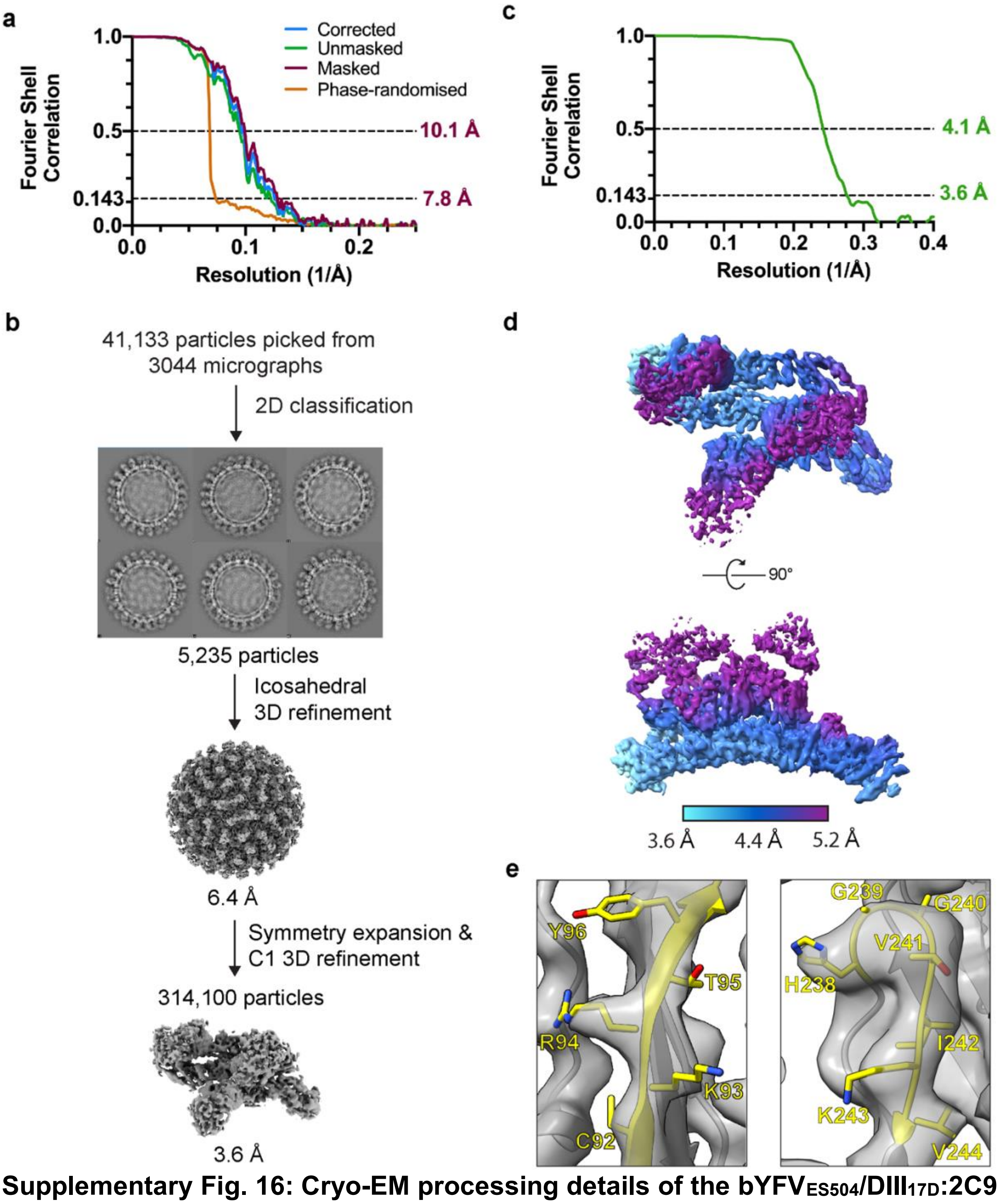

Supplementary Fig. 16: Cryo-EM processing details of the bYFV$_{ES504}$/DIII$_{17D}$:2C9 reconstructions. **a** FSC plot of the bYFV$_{ES504}$/DIII$_{17D}$:2C9 reconstruction. **b** Flowchart of single particle analysis performed on *cis*TEM2 to reach the final cryo-EM reconstruction of bYFV$_{ES504}$/DIII$_{17D}$:2C9 ASU. Displayed resolution values were calculated with half-map FSC$_{0.143}$ criterion. **c** Part. FSC curve of bYFV$_{ES504}$/DIII$_{17D}$:2C9 ASU, calculated using a loose spherical mask on *cis*TEM2. **d** Density map of bYFV$_{ES504}$/DIII$_{17D}$ ASU coloured according to its local resolution. Local resolution values were calculated using RELION 3.1.3 with *cis*TEM2 generated half maps. **e** High resolution detail of the bYFV$_{ES504}$/DIII$_{17D}$ ASU atomic model fitted in the cryo-EM ASU density map of bYFV$_{ES504}$/DIII$_{17D}$:2C9. Source data has been provided as a Source Data file.

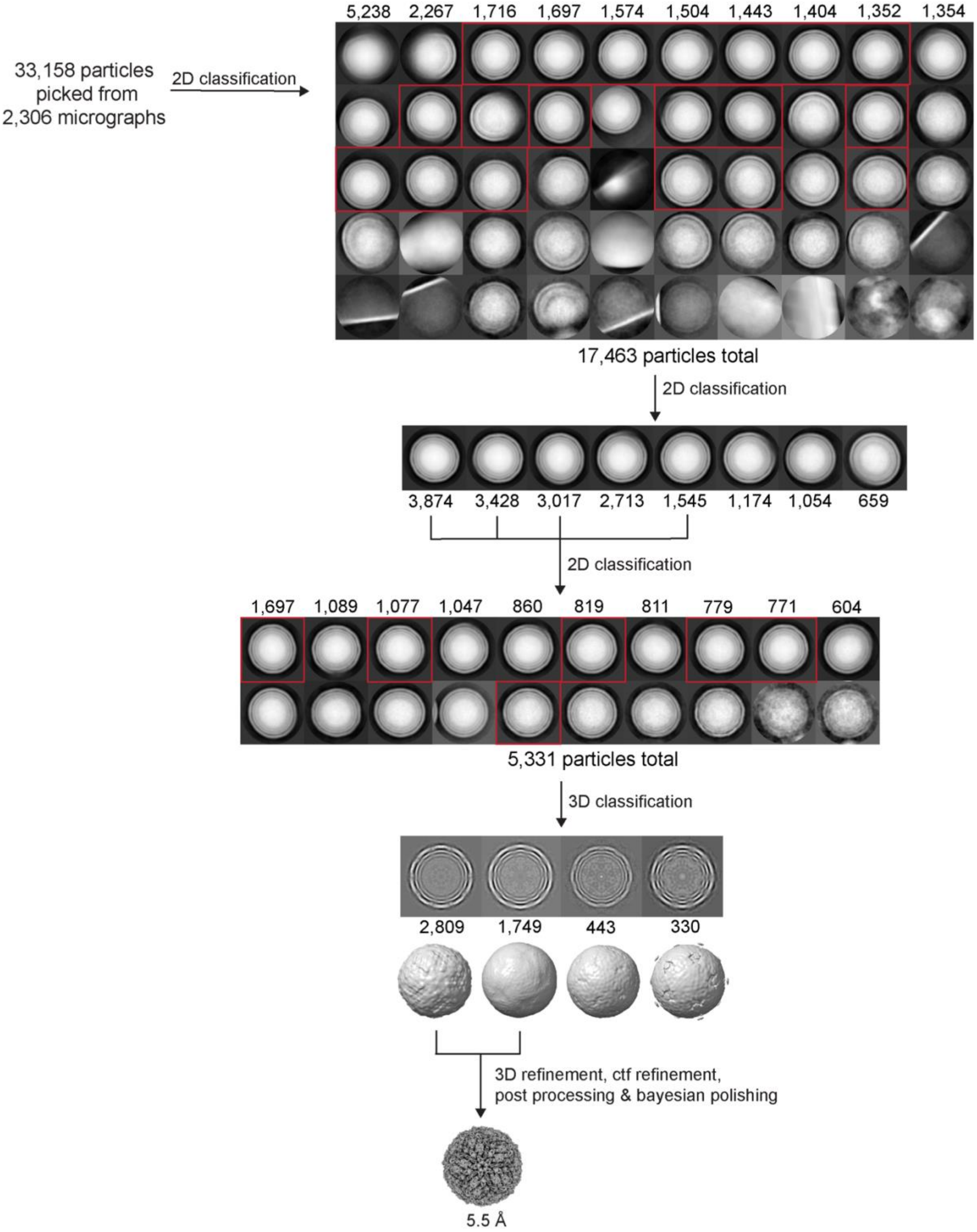

Supplementary Fig. 17: Cryo-EM processing flowchart of bYFV$_{Asibi}$/DIII$_{17D}$. Flowchart of single particle analysis steps performed on RELION 3.1.3 to reach the final 3D reconstruction of bYFV$_{Asibi}$/DIII$_{17D}$. Displayed resolution values were calculated with half-map FSC$_{0.143}$ criterion.

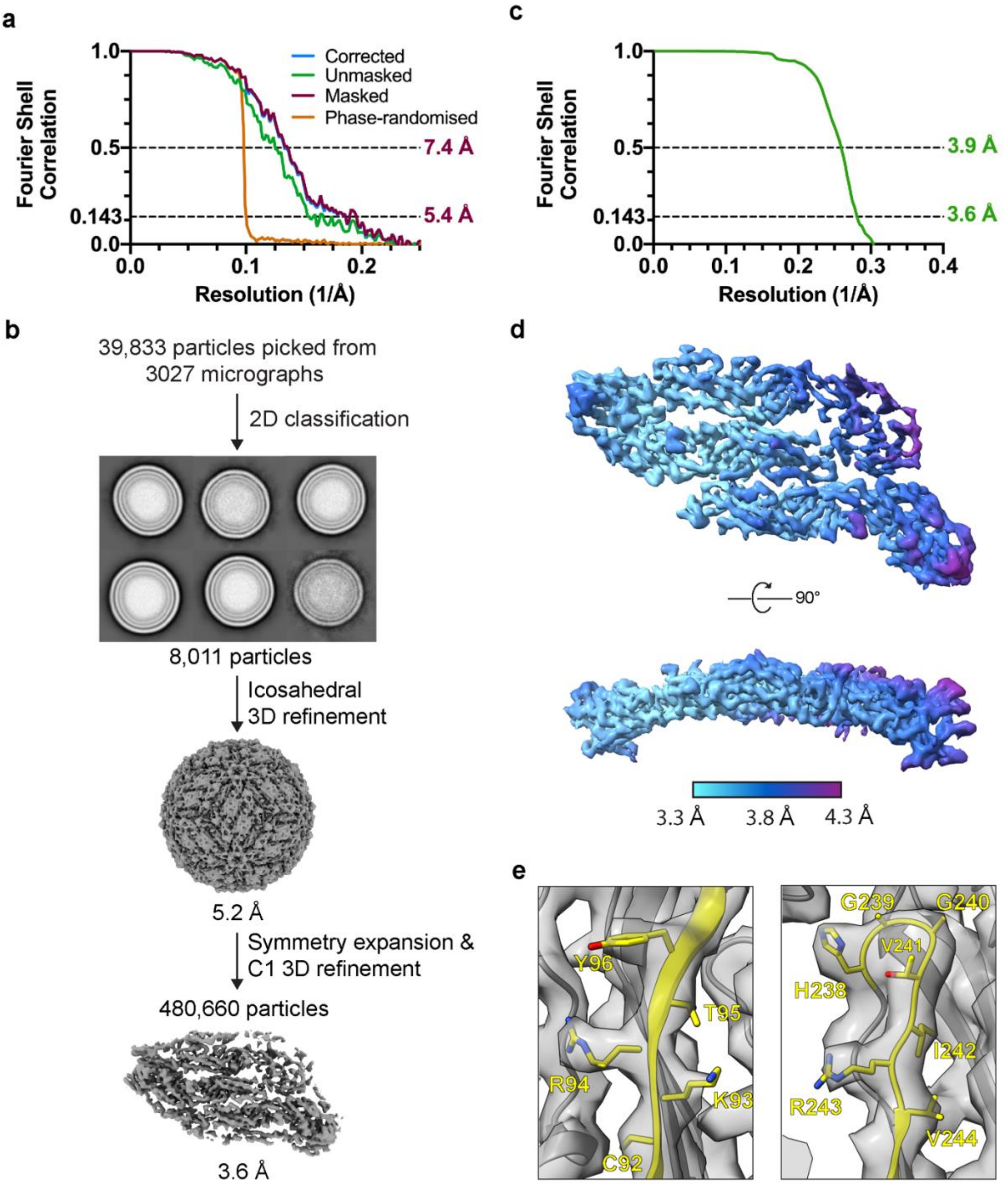

Supplementary Fig. 18: Cryo-EM processing details of bYFV$_{Asibi}$/DIII$_{17D}$ and bYFV$_{Asibi}$/DIII$_{17D}$ ASU reconstructions. a FSC plot of the bYFV$_{Asibi}$/DIII$_{17D}$ reconstruction. **b** Flowchart of single particle analysis performed on *cis*TEM2 to reach the final cryo-EM reconstruction of bYFV$_{Asibi}$/DIII$_{17D}$ ASU. Displayed resolution values were calculated with half-map FSC$_{0.143}$ criterion. **c** Part. FSC curve of bYFV$_{Asibi}$/DIII$_{17D}$ ASU, calculated using a loose spherical mask on *cis*TEM2. **d** Density map of bYFV$_{Asibi}$/DIII$_{17D}$ ASU coloured according to its local resolution. Local resolution values were calculated using RELION 3.1.3 with *cis*TEM2 generated half maps. **e** High resolution detail of the bYFV$_{Asibi}$/DIII$_{17D}$ ASU atomic model fitted in the cryo-EM density map. Source data has been provided as a Source Data file.

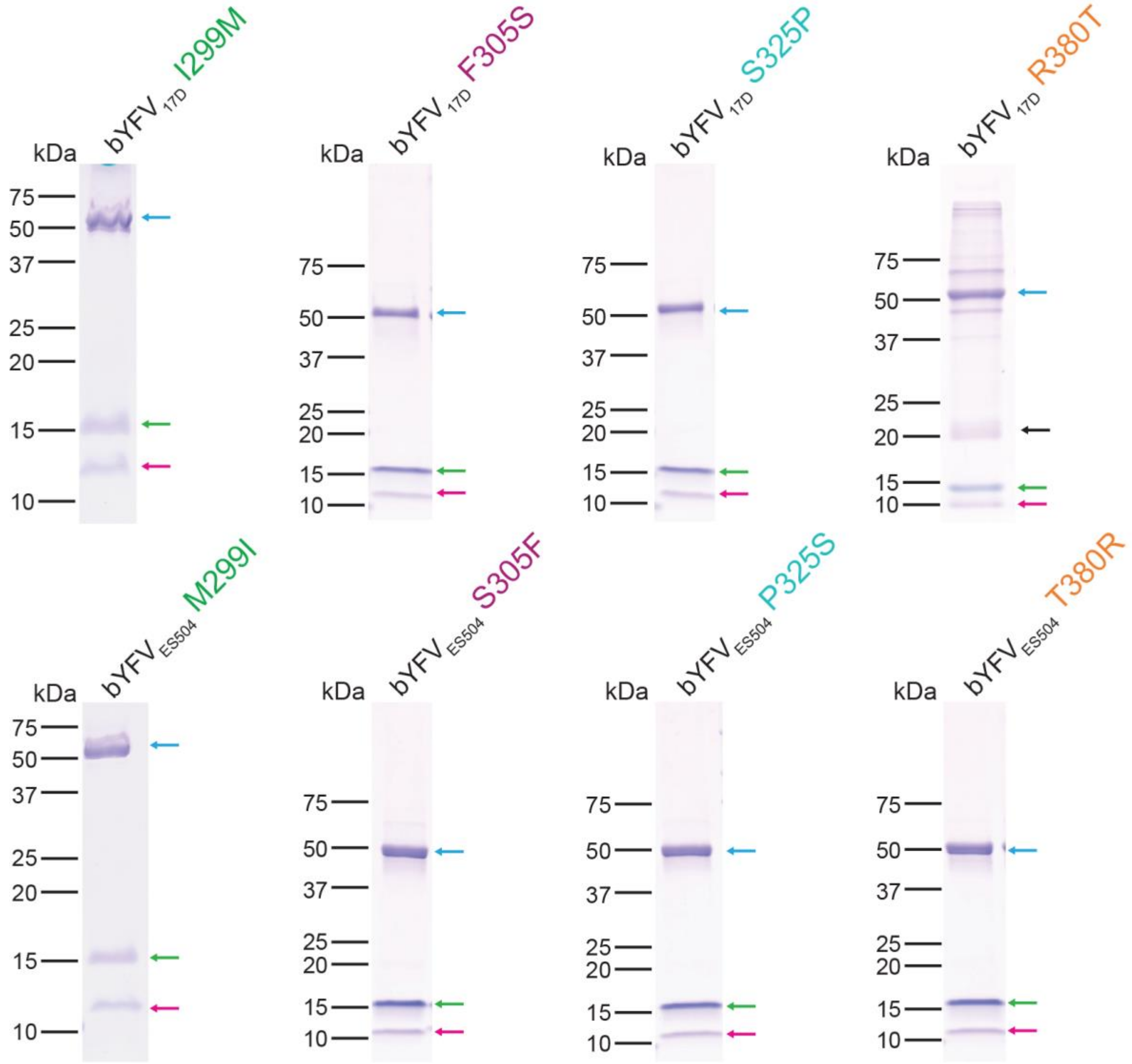

Supplementary Fig. 19: SDS-PAGE of bYFV DIII mutant chimeras. Purified viruses (10 μg) were separated by SDS-PAGE under reducing conditions and stained with Coomassie Blue. Envelope, pre-membrane, capsid and membrane proteins are indicated by blue, black, green and pink arrows, respectively. Source data has been provided as a Source Data file.

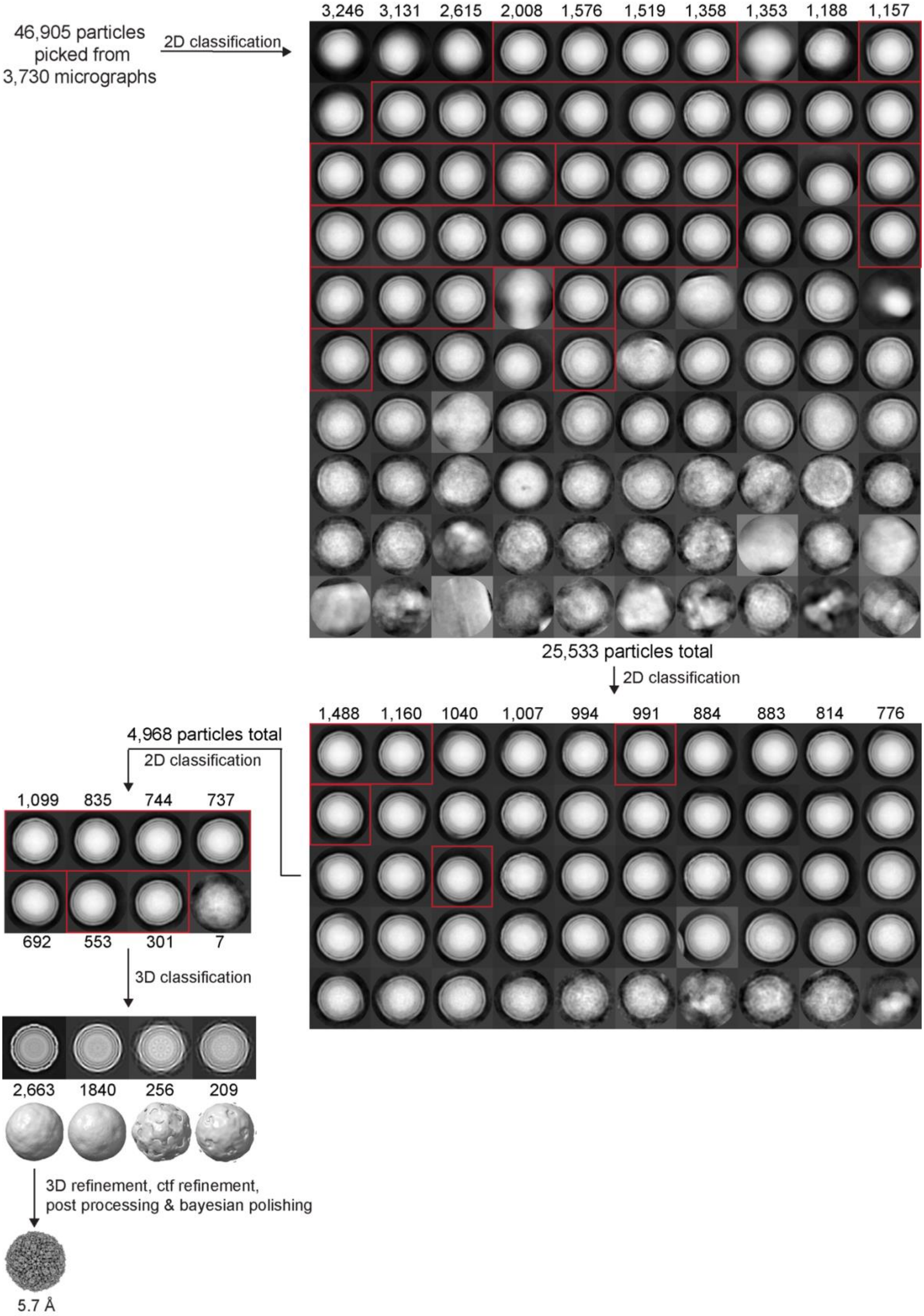

Supplementary Fig. 20: Cryo-EM processing flowchart of bYFV$_{ES504}$ T380R. Flowchart of single particle analysis steps performed on RELION 3.1.3 to reach the final 3D reconstruction of bYFV$_{ES504}$ T380R. Displayed resolution values were calculated with half-map FSC$_{0.143}$ criterion.

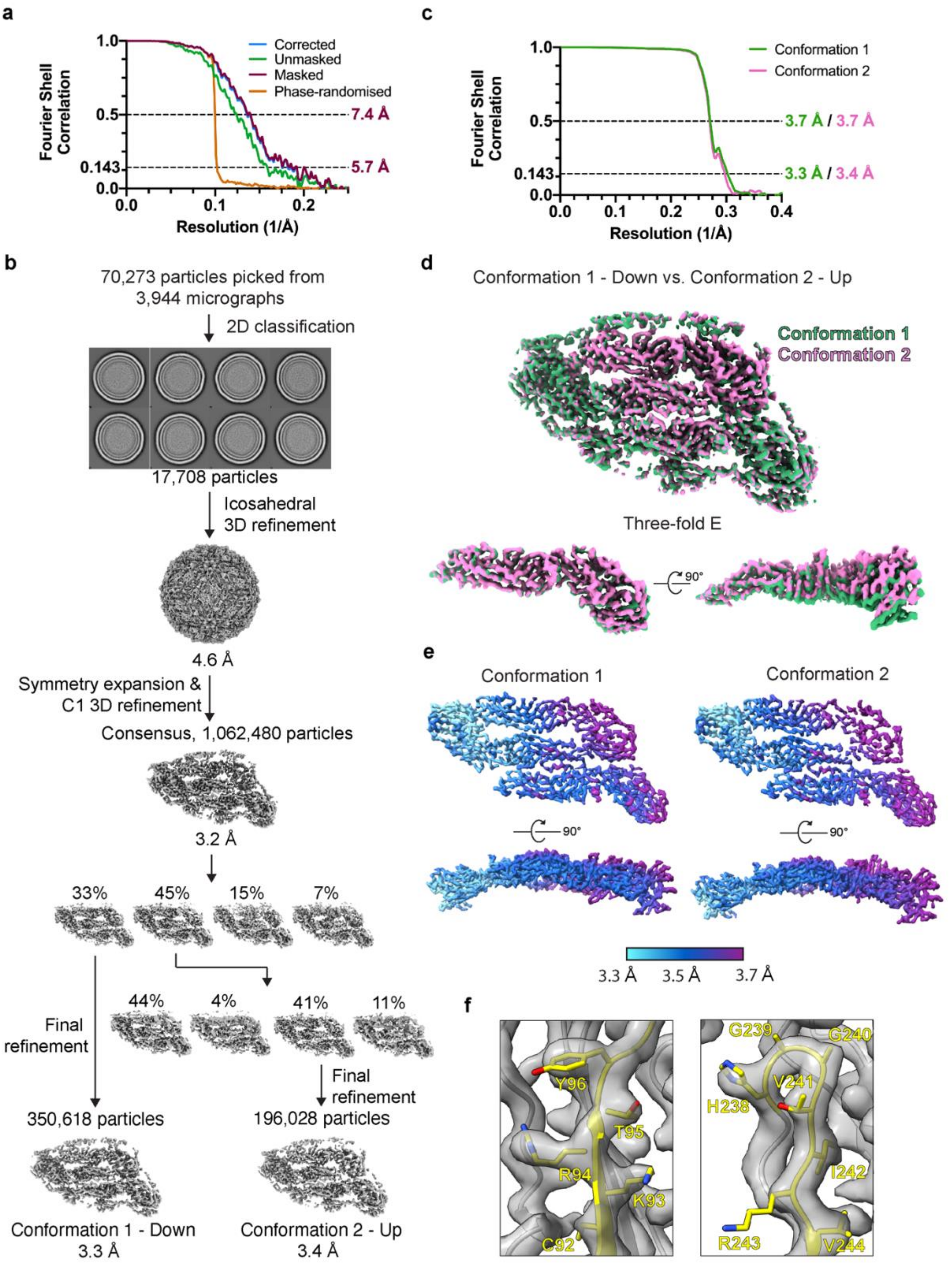

Supplementary Fig. 21: Cryo-EM details of the bYFV$_{ES504}$ T380R reconstructions. **a** FSC plot of the bYFV$_{ES504}$ T380R cryo-EM reconstruction. **b** Flowchart of single particle analysis performed on *cis*TEM2 to reach the final cryo-EM reconstructions of bYFV$_{ES504}$ T380R ASU. 3D classifications of the ASU conformations were performed on RELION 3.1.3. Displayed resolution values were calculated with half-map FSC$_{0.143}$ criterion. **c** Part. FSC curve of both bYFV$_{ES504}$ T380R ASU conformations, calculated using loose spherical masks on *cis*TEM2. **d** Comparison of the bYFV$_{ES504}$ T380R ASU

conformations. **e** Density map of both bYFV$_{ES504}$ T380R ASU conformations coloured according to their local resolution. Local resolution values were calculated using RELION 3.1.3 with *cis*TEM2 generated half maps. **f** High resolution detail of the bYFV$_{ES504}$ T380R ASU conformation 1 atomic model fitted in the cryo-EM ASU density map. Source data has been provided as a Source Data file.

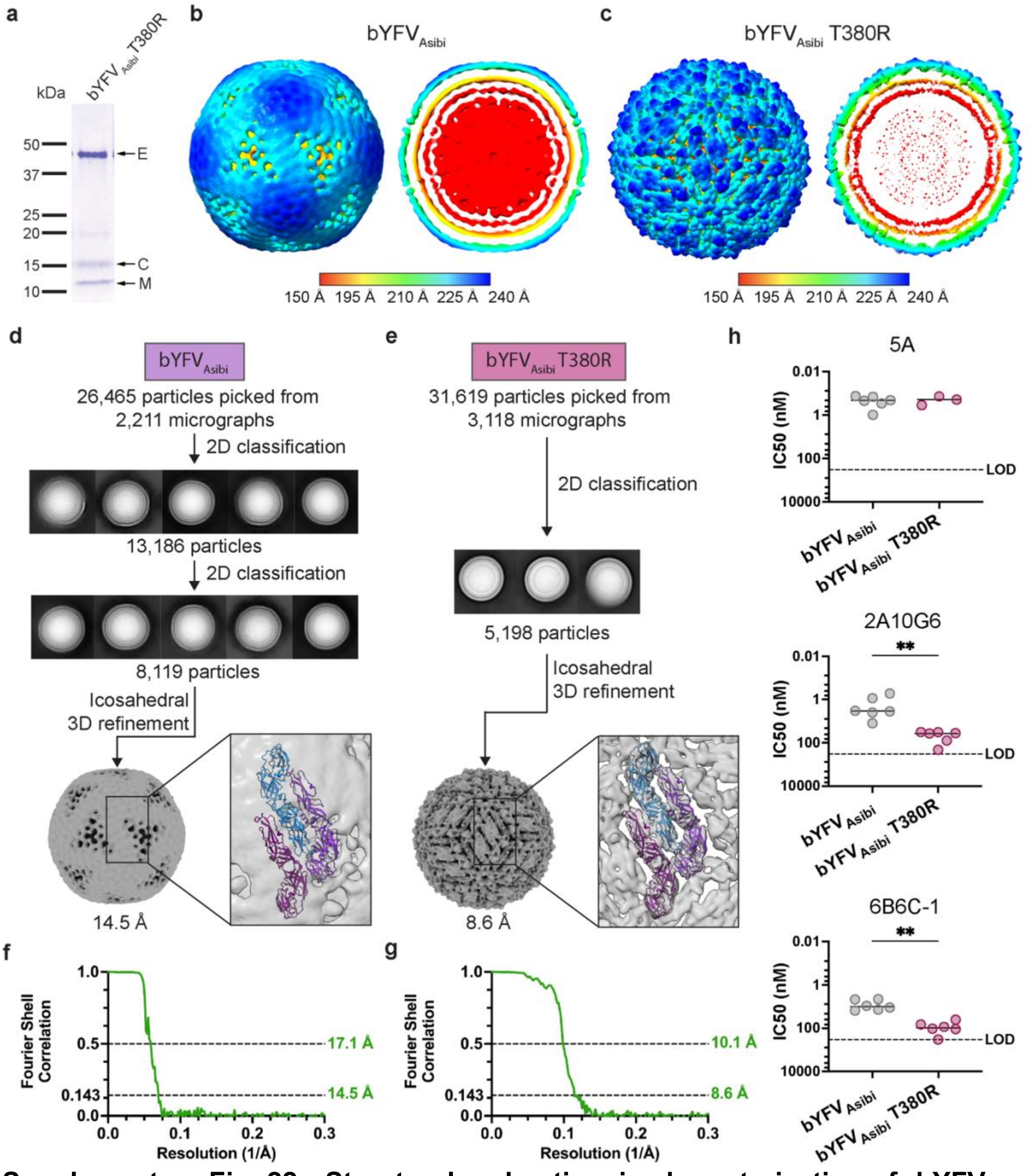

Supplementary Fig. 22: Structural and antigenic characterisation of bYFV$_{Asibi}$ and bYFV$_{Asibi}$ T380R. a Purified bYFV$_{Asibi}$ T380R (10 μg) was separated by SDS-PAGE under reducing conditions and stained with Coomassie Blue. Viral protein identity indicated by arrows. **b-c** Cryo-EM reconstruction of bYFV$_{Asibi}$ T380R (**b**) and bYFV$_{Asibi}$ (**c**) with I symmetry applied. Maps are radially coloured according to the following: 0-150 Å red, 151-195 Å yellow, 196-210 Å green, 211-225 Å cyan, 226-240 Å blue Å. **d-e** Flowchart of single particle analysis performed on *cis*TEM2 to reach the final cryo-EM reconstructions of bYFV$_{Asibi}$ T380R (**d**) and bYFV$_{Asibi}$ (**e**). The initial 3D reconstructions of the icosahedral virion were carried out *ab initio* in *cis*TEM2. In (d-e), the inset depicts the bYFV$_{17D}$ ASU model rigid-fit into the corresponding cryo-EM reconstruction. **f-g** Part. FSC curve of the bYFV$_{Asibi}$ T380R (**f**) and bYFV$_{Asibi}$ (**g**) reconstructions, calculated using loose spherical masks on *cis*TEM2. **h** IC50 values of recombinant hIgG1 anti-YFV or anti-flavivirus mAbs against the bYFV$_{Asibi}$ T380R.

Neutralisation was determined via FRNTs on C6/36 (*Aedes albopictus*) cells. Each symbol represents a technical replicate from three biological replicates (n = 6 or 3) and lines indicate group medians. Parental $bYFV_{Asibi}$ controls (in grey) are from Figure 1E. bYFV T380R was compared to $bYFV_{Asibi}$ using Mann-Whitney tests on GraphPad Prism 9. ** $p = 0.002$ and LOD = limit of detection. Source data has been provided as a Source Data file.

Supplementary Table 1. Refinement statistic details for $bYFV_{17D}$ and $bYFV_{ES504}$ reconstructions. Related to Figure 1 and 2.

	$bYFV_{17D}$	$bYFV_{ES504}$	$bYFV_{17D}$:5A	$bYFV_{ES504}$:5A	$bYFV_{17D}$:2C9	$bYFV_{ES504}$:2C9
**Data collection and processing**						
Magnification	50,000	50,000	50,000	50,000	50,000	50,000
Voltage (kV)	300	300	300	300	300	300
Frames per movie	50	50	50	50	50	50
Total dose (e^-/Å^2)	54.25	51.54	59.14	59.14	51.38	54.25
Nominal pixel size (Å)	0.96	0.96	0.96	0.96	0.96	0.96
Final pixel size (Å)	0.96	0.96	0.96	0.96	0.96	0.96
Defocus (µm)	-2.0	-1.5 to -2.5	-1.5 to -2.5	-1.5 to -2.5	-1.5 to -2.5	-1.5 to -2.5
EMDB	EMD-44278	EMD-44279	EMD-44282	EMD-44283	EMD-44280	EMD-44281
Micrographs	754	985	650	322	356	373
Particles	3,300	18,450	3,239	3,999	1,751	1,012
Box size (pixels)	640	640	750	750	750	750
Symmetry	I3	I3	I3	I3	I3	I3
SPA Software	RELION3.1.3	RELION3.1.3	RELION3.1.3	RELION3.1.3	RELION3.1.3	RELION3.1.3
**Fitting**						
Resolution at 0.143 FSC (Å)	7.5	12.6	11.8	15.5	7.3	21.8

Supplementary Table 2. Donor Information. Related to Figure 1.

Donor	Age	Sex	Year of vaccination	Year of 2nd vaccination	Blood drawn
1	36	F	9	-	2021
2	45	F	2007	2017	2021
3	45	M	2002	-	2021
4	30	M	2018	-	2021
5	41	M	2002	-	2021
6	44	M	2011	-	2021
7	49	M	2013	-	2021
8	32	F	2019	-	2021
9	32	F	2018	-	2021
10	45	F	2003	2011	2021
11	32	M	2007	-	2021
12	44	M	2008	-	2021
13	37	F	2002	-	2021
14	27	M	2019	-	2021

Supplementary Table 3. Refinement and model statistics for the ASU reconstructions. Related to Fig. 2, 4 and 6.

	**$bYFV_{17D}$:2C9 ASU**	**$bYFV_{ES504}/DIII_{17D}$ ASU**	**$bYFV_{ES504}/DIII_{17D}$:2C9 ASU**	**bYFVAsibi/$DIII_{17D}$ ASU**	**$bYFV_{ES504}$ T380R ASU**
**Data collection and processing**					
Magnification	50,000	60,000	60,000	60,000	60,000
Voltage (kV)	300	300	300	300	300
Frames per movie	50	40	40	40	40
Total dose ($e^-/Å^2$)	51.38	40	40	40	40
Nominal pixel size (Å)	0.96	0.4	0.4	0.4	0.4
Final pixel size (Å)	0.96	0.77	0.962	1.55	0.8
Defocus (µm)	-1.5 to -2.5	-0.5 to -2.5	-0.5 to -2.5	-0.5 to -2.5	-0.5 to -2.5
PDB/EMDB	9B6U/EMD-44288	9B6V/EMD-44289	9B6W/EMD-44290	9B6X/EMD-44291	9B6Y/EMD-44292
Micrographs	393	3029	3044	3027	3944
Particles	560,280	800,460	314,100	480,660	350,618
Box size (pixels)	360	440	440	250	440
Symmetry	C1	C1	C1	C1	C1
Map Resolution at 0.143 FSC (Å)	4.0	3.3	3.6	3.6	3.3
SPA Software	*cis*TEM2	*cis*TEM2	*cis*TEM2	*cis*TEM2	*cis*TEM2
**Fitting**					
Initial model used	$bYFV_{ES504}$ T380R	$bYFV_{ES504}$ T380R	$bYFV_{ES504}/DIII_{17D}$	$bYFV_{ES504}$ T380R	Model Angelo
Model Resolution (Å)	4.2	3.5	3.6	3.6	1.9
FSC threshold	0.143	0.143	0.143	0.143	0.143
Cross-correlation (masked)	0.78	0.83	0.72	0.74	0.75
Map-sharpening *B* factor ($Å^2$)	-90	-90	-90	-90	-90
Model composition					
Nonhydrogen	14,385	8,976	14,307	8,895	8,955
Protein residues	1,854	1,173	1,854	1,173	1,173
Ligands	NAG: 3	-	-	-	-

B factors ($Å^2$)					
Protein	157.44	90.31	226.96	162.71	81.97
Ligand	175.34	-	-	-	-
R.m.s deviations					
Bond lengths (Å)	0.009	0.011	0.006	0.005	0.008
Bond angles (°)	1.062	0.936	1.010	0.948	0.757
Validation					
MolProb score	1.21	1.16	1.4	1.06	1.57
Clash score	1.90	1.74	2.22	0.84	3.77
Poor rotamers (%)	0.06	0.00	0.00	0.00	
Ramachandran plot					
Favoured (%)	96.12	96.49	93.95	95.63	93.92
Allowed (%)	3.81	3.51	6.05	4.37	5.83
Disallowed (%)	0.00	0.00	0.00	0.00	0.00

Supplementary Table 4. Data collection and refinement statistics of the 2C9 Fab Crystal Structure. Related to Supplementary Fig. 9.

	2C9 Fab
**Data collection**	
Resolution Range (Å) (high)	49.5-2.66 (2.75-2.66)
Space group	$P2_1$
Unit Cell (Å)	67.91, 125.80, 97.24
Unit cell (º)	90.00, 93.93, 90.00
Total reflections	329663 (32993)
Unique reflections	46392 (4537)
Multiplicity	7.1 (7.3)
Completeness	100.0 (99.9)
Mean I / sigma (I)	8.3 (1.5)
R-merge	0.321 (2.914)
R-pim	0.129 (1.163)
Reflections used in refinement	46334
Reflections used for R-free	2328 (4.94%)
PDB	9B8G
**Refinement Statistics**	
Resolution Range (Å) (high)	49.5-2.66 (2.72-2.66)
Reflections used in refinement	46334 (2748)
Reflections used for R-free	2328 (149)
R-work (%)	20.08 (29.01)
R-free (%)	25.84 (32.38)
Number of non-hydrogen atoms	10200
Macromolecules	9922
Ligands	31
Solvent	247
R-free (%)	25.84 (32.38)
RMSD (bonds)	0.005

RMSD (angles)	0.74
Ramachandran favoured (%)	97.97
Ramachandran outliers (%)	0.0
Rotamer outliers	0.18
Clashscore	4.31
Average B-factor	47.11
Macromolecules	47.15
Ligands	57.94
Solvent	44.00

Supplementary Table 5. Refinement statistics for the bYFV prME chimera and bYFV single mutant reconstructions. Related to Figure 4, 6 and Supplementary Fig. 22.

	**$bYFV_{ES504}/DIII_{17D}$**	**$bYFV_{ES504}/DIII_{17D}$:2C9**	**$bYFV_{Asibi}/DIII_{17D}$**	**$bYFV_{ES504}$ T380R**	**$bYFV_{Asibi}$**	**$bYFV_{Asibi}$ T380R**
**Data collection and processing**						
Magnification	60,000	60,000	60,000	60,000	60,000	60,000
Voltage (kV)	300	300	300	300	200	200
Frames per movie	40	40	40	40	40	40
Total dose ($e^-/Å^2$)	40	40	40	40	40	40
Nominal pixel size (Å)	0.4	0.4	0.4	0.4	0.848	0.848
Final pixel size (Å)	0.8	1.6	0.8	0.8	0.87	0.87
Defocus (μm)	-0.5 to -2.5	-0.5 to -2.5	-0.5 to -2.5	-0.5 to -2.5	-0.5 to -2.5	-0.5 to -2.5
EMDB	EMD-44284	EMD-44285	EMD-44286	EMD-44287	EMD-49804	EMD-49805
Micrographs	1,672	2,234	2,306	3,730	2,211	3,118
Particles	3,325	1,376	4,558	2,663	8,119	5,198
Box size (pixels)	768	432	768	768	768	768
Symmetry	I3	I3	I3	I3	I	I
SPA Software	RELION3.1.3	RELION3.1.3	RELION3.1.3	RELION3.1.3	*cis*TEM2	*cis*TEM2
**Fitting**						
Resolution at 0.143 FSC (Å)	6.5	8.1	5.5	5.7	14.5	8.6
