# Supplementary material for: A single residue in the yellow fever virus envelope protein modulates virion architecture and antigenicity
Source: Nat Commun. 2025 Sep 26;16:8449. doi: 10.1038/s41467-025-63038-5 (PMC12475062; doi:10.1038/s41467-025-63038-5)
Supplement: Supplementary file 2 — Description Of Additional Supplementary File [file 41467_2025_63038_MOESM2_ESM.pdf]

Description of additional supplementary files

Supplementary data 1:

List of primers used to generate chimeric viruses
