# Supplementary material for: A single residue in the yellow fever virus envelope protein modulates virion architecture and antigenicity
Source: Nat Commun. 2025 Sep 26;16:8449. doi: 10.1038/s41467-025-63038-5 (PMC12475062; doi:10.1038/s41467-025-63038-5)
Supplement: Supplementary file 4 — Reporting summary [file 41467_2025_63038_MOESM4_ESM.pdf]

nature portfolio

Corresponding author(s): Daniel Watterson

Last updated by author(s): Jul 28, 2025

Reporting Summary

Nature Portfolio wishes to improve the reproducibility of the work that we publish. This form provides structure for consistency and transparency in reporting. For further information on Nature Portfolio policies, see our Editorial Policies and the Editorial Policy Checklist.

Statistics

For all statistical analyses, confirm that the following items are present in the figure legend, table legend, main text, or Methods section.

n/a	Confirmed	
☐	☒	The exact sample size (*n*) for each experimental group/condition, given as a discrete number and unit of measurement
☐	☒	A statement on whether measurements were taken from distinct samples or whether the same sample was measured repeatedly
☐	☒	The statistical test(s) used AND whether they are one- or two-sided<br>*Only common tests should be described solely by name; describe more complex techniques in the Methods section.*
☐	☒	A description of all covariates tested
☐	☒	A description of any assumptions or corrections, such as tests of normality and adjustment for multiple comparisons
☐	☒	A full description of the statistical parameters including central tendency (e.g. means) or other basic estimates (e.g. regression coefficient) AND variation (e.g. standard deviation) or associated estimates of uncertainty (e.g. confidence intervals)
☐	☒	For null hypothesis testing, the test statistic (e.g. F, t, r) with confidence intervals, effect sizes, degrees of freedom and P value noted<br>*Give P values as exact values whenever suitable.*
☒	☐	For Bayesian analysis, information on the choice of priors and Markov chain Monte Carlo settings
☒	☐	For hierarchical and complex designs, identification of the appropriate level for tests and full reporting of outcomes
☒	☐	Estimates of effect sizes (e.g. Cohen's *d*, Pearson's *r*), indicating how they were calculated

Our web collection on statistics for biologists contains articles on many of the points above.

Software and code

Policy information about availability of computer code

Data collection	SerialEM (v. 3.1)
Data analysis	RELION (v. 3.1.3), cisTEM (v. 2.0), symmetry_expand_stack_and_par, MotionCor2 (v. 1.1.0), CTFFIND (v. 4.1), DeepEMhancer, ChimeraX (v. 1.2.5), COOT (v. 0.8.9.2), ISOLDE (v. 1.2.1), PHENIX (v. 1.19.2), ModelAngelo (v. 1.0), GraphPad Prism (v. 9.1.0).

For manuscripts utilizing custom algorithms or software that are central to the research but not yet described in published literature, software must be made available to editors and reviewers. We strongly encourage code deposition in a community repository (e.g. GitHub). See the Nature Portfolio guidelines for submitting code & software for further information.

Data

Policy information about availability of data

All manuscripts must include a data availability statement. This statement should provide the following information, where applicable:

- Accession codes, unique identifiers, or web links for publicly available datasets
- A description of any restrictions on data availability
- For clinical datasets or third party data, please ensure that the statement adheres to our policy

The cryo-EM/X-ray crystallography maps and models generated in this study have been deposited in the Electron Microscopy Data Bank and the Protein Data Bank, respectively, under accession codes: bYFV17D (EMD-44278), bYFVES504 (EMD-44279), bYFV17D:2C9 (EMD-44280), bYFVES504:2C9 (EMD-44281), bYFV17D:5A (EMD-44282), bYFVES504:5A (EMD-44283), bYFVES504/DIII17D (EMD-44284), bYFVES504/DIII17D:2C9 (EMD-44285), bYFVAsibi/DIII17D (EMD-44286), bYFVES504

T380R (EMD-44287), bYFVAsibi (EMD-44904), bYFVAsibi T380R (EMD-49805), bYFV17D:2C9 ASU (EMD-44288, PDB: 9B6U), bYFVES504/DIII17D ASU (EMD-44289, PDB: 9B6V), bYFVES504/DIII17D:2C9 (EMD-44290, PDB: 9B6W), bYFVAsibi/DIII17D ASU (EMD-44291, PDB: 9B6X), bYFVES504 T380R ASU (EMD-44292, PDB: 9B6Y) and 2C9 Fab (PDB: 9B8G). Other structures used in this study are available in the Protein Database under accession codes 6IW4 (X-ray crystal structure of YFV17D) and 7JVD (Fab of 5.6 mAb). Source data are provided with this paper.

Research involving human participants, their data, or biological material

Policy information about studies with human participants or human data. See also policy information about sex, gender (identity/presentation), and sexual orientation and race, ethnicity and racism.

Reporting on sex and gender	Sex was determined based on self-reporting. Results are not specific to a sex or gender.
Reporting on race, ethnicity, or other socially relevant groupings	Race, ethnicity or other social grouping not relevant to study.
Population characteristics	Human volunteers were between 27 and 49 years of age when samples were taken. Of the 14 individuals, 8 were males and 6 were females.
Recruitment	Participants were recruited on a volunteer basis. Participants provided written informed consent prior to participation.
Ethics oversight	Ethics approved by The University of Queensland Human Ethics Unit.

Note that full information on the approval of the study protocol must also be provided in the manuscript.

Field-specific reporting

Please select the one below that is the best fit for your research. If you are not sure, read the appropriate sections before making your selection.

☒ Life sciences ☐ Behavioural & social sciences ☐ Ecological, evolutionary & environmental sciences

For a reference copy of the document with all sections, see nature.com/documents/nr-reporting-summary-flat.pdf

Life sciences study design

All studies must disclose on these points even when the disclosure is negative.

Sample size	Cryo-EM sample size was not predetermined. Each cryo-EM dataset contained thousands of particles. The size of each dataset was based on instrument availability. Relevant particle numbers for each dataset are provided in the Supplementary Figures and Tables file. The sample size of the human sera sample was determined by the availability of volunteers with a history of YFV vaccination.
Data exclusions	Cryo-EM images of poor quality were removed based on assessment of the contrast transfer function. Severly disordered particles were removed from the datasets throughout 2D and 3D classification. Please refer to the processing workflows in the Supplementary Figures and Table file.
Replication	All attempts have been made to verify the reproducibility of the results. Independent biological replicates and technical replicates were performed for the FRNTs. Majority of the virus preparations were purified, plunge frozen and imaged via cryo-EM more then once. Large cryo-EM datasets for further processing was performed once for each dataset.
Randomization	Cryo-EM data was randomised into two half-datasets and independently refined. Fourier Shell Correlation between two half-datasets was used to provide a "gold-standard" estimate of the resolution of the cryo-EM map. These are provided in the Supplementary Figures and Table file.
Blinding	Blinding is not relevant. Analysis and collection of cryo-EM data did not require statistical interpretation and human bias is mitigated by established validation metrics.

Reporting for specific materials, systems and methods

We require information from authors about some types of materials, experimental systems and methods used in many studies. Here, indicate whether each material, system or method listed is relevant to your study. If you are not sure if a list item applies to your research, read the appropriate section before selecting a response.

Materials & experimental systems

n/a	Involved in the study
☐	☒ Antibodies
☐	☒ Eukaryotic cell lines
☒	☐ Palaeontology and archaeology
☒	☐ Animals and other organisms
☒	☐ Clinical data
☒	☐ Dual use research of concern
☒	☐ Plants

Methods

n/a	Involved in the study
☒	☐ ChIP-seq
☒	☐ Flow cytometry
☒	☐ MRI-based neuroimaging

Antibodies

Antibodies used

5A (Daffis et al. 2005), 2C9 (Thibodeaux et al. 2012), 864 (Calvert et al. 2016), 2A10G6 (Dai et al. 2016), 6B6C-1 (Thibodeaux et al. 2009), 4G4 (Clark et al. 2007), IRDye® 800CW goat anti-mouse IgG secondary antibody (LI-COR Biosciences, Cat # 926-32210, Lot #D40312-15), Goat anti-human IgG Fc highly cross-adsorbed secondary antibody HRP (Invitrogen, Cat # A18829, Lot #95-14-052722). 5A, 2C9, 864, 2A10G6 and 6B6C-1 were used at a starting concentration of 50 ug/mL. 4G4 was used at 1 ug/mL and both secondary antibodies were used at 0.2 ug/mL.

Validation

5A: Daffis et al. Virology. 2005. 337, 262-272, doi:https://doi.org/10.1016/j.virol.2005.04.031.
2C9: Thibodeaux et al. Antiviral Res. 2012. 94, 1-8, doi:10.1016/j.antiviral.2012.02.001.
864: Calvert et al. Antiviral Res. 2016. 131, 92-99, doi:10.1016/j.antiviral.2016.04.013.
2A10G6: Dai et al. Cell Host Microbe. 2016. 19, 696-704, doi:https://doi.org/10.1016/j.chom.2016.04.013.
6B6C-1: Thibodeaux & Roehrig. Clin Vaccine Immunol. 2009. 16, 679-685, doi:10.1128/cvi.00354-08.
4G4: Clark et al. J Gen Virol. 2007. 88, 1175-1183, doi:10.1099/vir.0.82609-0.

Eukaryotic cell lines

Policy information about cell lines and Sex and Gender in Research

Cell line source(s)

C6/36 (Aedes albopictus, ATCC – CL1660)
ExpiCHO cells (Gibco, Cat # A29127)

Authentication

Cell lines were not genetically confirmed but were visually confirmed.

Mycoplasma contamination

Cell lines tested negative for mycoplasma.

Commonly misidentified lines
(See ICLAC register)

No commonly misidentified cell lines were used in this study.

Plants

Seed stocks

N/A

Novel plant genotypes

N/A

Authentication

N/A
